# Characterising sources of PM_2·5_ exposure for school children with asthma: a personal exposure study across six cities in sub-Saharan Africa

**DOI:** 10.1016/S2352-4642(23)00261-4

**Published:** 2024-01

**Authors:** Shanon Lim, Bibie Said, Lindsay Zurba, Gioia Mosler, Emmanuel Addo-Yobo, Olayinka Olufunke Adeyeye, Bernard Arhin, Dimitris Evangelopoulos, Victoria Temitope Fapohunda, Farida Fortune, Chris J Griffiths, Sbekezelo Hlophe, Marian Kasekete, Scott Lowther, Refiloe Masekela, Elizabeth Mkutumula, Blandina Theophil Mmbaga, Hilda Angela Mujuru, Rebecca Nantanda, Lovemore Mzati Nkhalamba, James S Ngocho, Oluwafemi Tunde Ojo, Sandra Kwarteng Owusu, Sunshine Shaibu, Ismail Ticklay, Jonathan Grigg, Benjamin Barratt

**Affiliations:** a MRC Centre for Environment and Health, Environmental Research Group, Imperial College London, London, UK; b Kilimanjaro Clinical Research Institute, Kilimanjaro Christian Medical Centre, Moshi, Tanzania; c Kibong'oto Infectious Disease Hospital, Hai, Tanzania; d Kilimanjaro Christian Medical University College, Moshi, Tanzania; e Department of Civil and Environmental Engineering, Faculty of Engineering, The University of Auckland, Auckland, New Zealand; f Education for Health Africa, Durban, South Africa; g Centre for Genomics and Child Health, Blizard Institute, Faculty of Medicine and Dentistry, Queen Mary University of London, London, UK; h Department of Child Health, School of Medicine and Dentistry, Kwame Nkrumah University of Science and Technology, Kumasi, Ghana; i Department of Medicine, Lagos State University College of Medicine, and Lagos State University Teaching Hospital, Ikeja Lagos, Nigeria; j Komfo Anokye Teaching Hospital, Kumasi, Ghana; k Centre for Oral immunobiology and Regenerative Medicine, Faculty of Medicine and Dentistry, Queen Mary University of London, London, UK; l Asthma UK Centre for Applied Research, Wolfson Institute of Population Health, Faculty of Medicine and Dentistry, Queen Mary University of London, London, UK; m Department of Paediatrics and Child Health, Nelson R Mandela School of Clinical Medicine, College of Health Sciences, University of KwaZulu Natal, Durban, South Africa; n University of Zimbabwe Faculty of Medicine and Health Sciences, Harare, Zimbabwe; o Dyson Technology Limited, Malmesbury, Wiltshire, UK; p Malawi Liverpool Wellcome Programme, Blantyre, Malawi; q Makerere University Lung Institute, Makerere College of Health Sciences, Kampala Uganda; r NIHR NPRU in Environmental Exposures and Health, Imperial College London, London, UK

## Abstract

**Background:**

Air pollution is the second largest risk to health in Africa, and children with asthma are particularly susceptible to its effects. Yet, there is a scarcity of air pollution exposure data from cities in sub-Saharan Africa. We aimed to identify potential exposure reduction strategies for school children with asthma living in urban areas in sub-Saharan Africa.

**Methods:**

This personal exposure study was part of the Achieving Control of Asthma in Children in Africa (ACACIA) project. Personal exposure to particulate matter (PM) was monitored in school children in six cities in sub-Saharan Africa (Blantyre, Malawi; Durban, South Africa; Harare, Zimbabwe; Kumasi, Ghana; Lagos, Nigeria; and Moshi, Tanzania). Participants were selected if they were aged 12–16 years and had symptoms of asthma. Monitoring was conducted between June 21, and Nov 26, 2021, from Monday morning (approximately 1000 h) to Friday morning (approximately 1000 h), by use of a bespoke backpack with a small air pollution monitoring unit with an inbuilt Global Positioning System (GPS) data logger. Children filled in a questionnaire detailing potential sources of air pollution during monitoring and exposures were tagged into three different microenvironments (school, commute, and home) with GPS coordinates. Mixed-effects models were used to identify the most important determinants of children's PM_2·5_ (PM <2·5 μm in diameter) exposure.

**Findings:**

330 children were recruited across 43 schools; of these, 297 had valid monitoring data, and 1109 days of valid data were analysed. Only 227 (20%) of 1109 days monitored were lower than the current WHO 24 h PM_2·5_ exposure health guideline of 15 μg/m^3^. Children in Blantyre had the highest PM_2·5_ exposure (median 41·8 μg/m^3^), whereas children in Durban (16·0 μg/m^3^) and Kumasi (17·9 μg/m^3^) recorded the lowest exposures. Children had significantly higher PM_2·5_ exposures at school than at home in Kumasi (median 19·6 μg/m^3^*vs* 14·2 μg/m^3^), Lagos (32·0 μg/m^3^*vs* 18·0 μg/m^3^), and Moshi (33·1 μg/m^3^*vs* 23·6 μg/m^3^), while children in the other three cities monitored had significantly higher PM_2·5_ exposures at home and while commuting than at school (median 48·0 μg/m^3^ and 43·2 μg/m^3^*vs* 32·3 μg/m^3^ in Blantyre, 20·9 μg/m^3^ and 16·3 μg/m^3^*vs* 11·9 μg/m^3^ in Durban, and 22·7 μg/m^3^ and 25·4 μg/m^3^*vs* 16·4 μg/m^3^ in Harare). The mixed-effects model highlighted the following determinants for higher PM_2·5_ exposure: presence of smokers at home (23·0% higher exposure, 95% CI 10·8–36·4), use of coal or wood for cooking (27·1%, 3·9–56·3), and kerosene lamps for lighting (30·2%, 9·1–55·2). By contrast, 37·2% (95% CI 22·9–48·2) lower PM_2·5_ exposures were found for children who went to schools with paved grounds compared with those whose school grounds were covered with loose dirt.

**Interpretation:**

Our study suggests that the most effective changes to reduce PM_2·5_ exposures in these cities would be to provide paving in school grounds, increase the use of clean fuel for cooking and light in homes, and discourage smoking within homes. The most efficient way to improve air quality in these cities would require tailored interventions to prioritise different exposure-reduction policies in different cities.

**Funding:**

UK National Institute for Health and Care Research.

## Introduction

Air pollution is one of the leading health risks, estimated to contribute to 6·7 million annual global deaths.^1^ Reducing the adverse health effects due to air pollution has been included in the UN's Sustainable Development Goals (SDGs), with aims to reduce deaths and illnesses from air pollution (SDG3.9) and improve air quality in cities (SDG11.6).^2^ The adverse health effects due to air pollution in Africa are of particular concern, since air pollution is the second largest risk factor for death for all ages on the continent after HIV, and this burden is expected to grow in the coming years.^3^ Despite this risk, there is a scarcity of regulatory air quality monitoring equipment to quantify exposure levels in sub-Saharan Africa, with continuous monitoring only conducted in three countries.^4^

**Research in context**

**Evidence before this study**

Air pollution is the second largest health risk in Africa, contributing to 1·1 million deaths in 2019, and children with asthma are particularly susceptible to its effects. Therefore, improving air quality is a targeted risk-reduction strategy in the UN Sustainable Developmental Goals. In sub-Saharan Africa, rapid urbanisation has been associated with deteriorating air quality, a situation that is expected to worsen in the coming years. Despite this, there is a dearth of human air pollution exposure data in sub-Saharan Africa. Our systematic review published in 2022 of studies recording personal exposure to PM_2·5_ (particulate matter <2·5 μm in diameter) found only nine of 111 studies were conducted in Africa. Only one small study to date has measured PM_2·5_ exposure in urban school children in Africa. Of the studies conducted in sub-Saharan Africa, the majority focused on rural areas and air pollution exposure from household air pollution. Therefore, there is uncertainty about the magnitude and sources of PM_2·5_ exposure to children in urban areas in sub-Saharan Africa. Such data are necessary to identify effective interventions to reduce the effects of air pollution on children with symptoms of asthma in cities in sub-Saharan Africa.

**Added value of this study**

As part of the Achieving Control of Asthma in Children in Africa (ACACIA) project, this study is, to the best of our knowledge, the largest personal PM_2·5_ exposure study and the first to be conducted in children with symptoms of asthma in urban centres in Africa. The study measured personal exposure for 297 school children with asthma in six cities in sub-Saharan Africa (Blantyre, Malawi; Durban, South Africa; Harare, Zimbabwe; Kumasi, Ghana; Lagos, Nigeria; and Moshi, Tanzania). This study used the latest high-resolution particulate matter mobile monitoring technology, with continuous 96 h exposure measurements of school children conducted with a monitor embedded in a school backpack. This amounted to 1109 days of exposure data. The study analysed GPS coordinates to identify microenvironments (home, school, and commute) where children's exposures were the highest. It also identified the potential contribution of natural and anthropogenic sources of this exposure by analysing PM_2·5_:PM_10_ ratios. Mixed-effects models were created to identify dominant determinants, facilitating the formulation of evidence-based recommendations to reduce exposure.

**Implications of all the available evidence**

The results of this study provide new information to inform recommendations on how PM_2·5_ exposure can be reduced in urban areas in sub-Saharan Africa to protect school children with respiratory diseases such as asthma. Although policies to improve air quality across all locations should be implemented, by using high time-resolution monitors to measure personal exposure we were able to identify the microenvironments in different cities where children's exposure to PM_2·5_ was highest, to guide prioritisation of intervention strategies. Most studies in Africa have focused on household air pollution, but this study reported high exposures at schools compared to home for children in three of the six cities monitored. This is likely to be due to resuspended dust from unpaved school grounds. These results highlight the need for more targeted interventions to rapidly reduce children's daily exposures to PM_2·5_ compared with generic policies for reducing air pollution.

Rapid urbanisation is predicted to continue in sub-Saharan Africa, with estimates highlighting that the urban population will increase from 43% of the total population in 2018 to 59% by 2050.^5^ Without intervention, this rapid urbanisation will have a detrimental impact on air quality.^4^ Fine particulate matter (ie, <2·5 μm in diameter; PM_2·5_) is a particularly scrutinised air pollutant because of its links with adverse health effects.^6^ Increased levels of PM_2·5_ exposure have been causally linked to respiratory diseases such as asthma,^7^ with higher susceptibility observed in children following both short-term and long-term exposure.^8^, ^9^ Furthermore, an increasing prevalence of asthma in Africa is thought to be due to increasing urbanisation on the continent.^10^ However, few studies to date have quantified the air pollution exposure of people in urban communities in Africa, with the majority of research focusing on household air pollution from biomass burning in rural communities.^4^, ^11^

Measurement of personal exposure with portable air pollution monitors has increased in the past decade as a result of advances in technology. Miniaturised monitors can provide high time-resolved measurements.^12^ These measurements, paired with participant global positioning system (GPS) coordinates, allow a better understanding of where, when, and how people are exposed to air pollution.^12^ This technology can provide exposure estimates for countries without regulatory monitors and allow for targeted interventions to reduce personal exposure.^4^

Our previous systematic review on personal PM_2·5_ exposure^11^ highlighted that despite low-income and middle-income countries having higher PM_2·5_ exposures than high-income countries, only four of 111 studies had been conducted in urban sub-Saharan Africa.^13^, ^14^, ^15^, ^16^ Therefore, there is an urgent need for more studies to identify the magnitude and sources of PM_2·5_ exposure in school children living in cities in sub-Saharan Africa. Although PM_2·5_ is the pollutant of interest, the ratio between PM_2·5_ and larger particles such as PM_10_ has been used to better identify the source of PM_2·5_ in different environments.^17^ Lower PM_2·5_:PM_10_ ratios indicate a higher proportion of larger particles and therefore have been associated with natural sources of PM_2·5_, such as resuspended dust, whereas higher ratios have been linked to anthropogenic sources.^17^, ^18^

This study aimed to characterise PM_2·5_ exposure in school children with asthma in six cities in sub-Saharan Africa. We used high-resolution continuous 96 h PM measurements combined with a novel analysis of GPS coordinates and PM_2·5_:PM_10_ ratios to characterise PM_2·5_ exposure by source and microenvironment. We aimed to identify potential exposure reduction interventions for school children living in urban areas in sub-Saharan Africa.

## Methods

### Study design and participants

This personal exposure study was a component of the Achieving Control of Asthma in Children in Africa (ACACIA) project, which aimed to understand and improve the health of young people with asthma in Africa.^19^ We monitored personal PM exposure in six cities in sub-Saharan Africa (Blantyre, Malawi; Durban, South Africa; Harare, Zimbabwe; Kumasi, Ghana; Lagos, Nigeria; and Moshi, Tanzania); 330 school children aged 12–16 years with asthma were recruited for this study. Briefly, research teams screened for and diagnosed asthma symptoms by conducting a breathing survey at schools, based on questions from the International Study of Asthma and Allergies in Childhood (ISAAC) asthma core questionnaire.^19^ Schools were invited to participate in the ACACIA study and those that agreed to participate were selected. Children identified during ACACIA screening with wheezing symptoms in the previous 12 months were invited to take part in this study.^19^ The site in Moshi, Tanzania, was added for the present study and did not participate in ACACIA. The ACACIA screening survey was therefore applied in schools in Moshi as part of this study. Monitoring at a seventh location (Kampala, Uganda) was discontinued because of COVID-19 pandemic restrictions. Children who were monitored provided assent to participate in the study and written signed consent was provided by their parents or guardians.

Monitoring was conducted between June 21, and Nov 26, 2021, during weekdays from Monday morning (approximately 1000 h) to Friday morning (approximately 1000 h) with up to eight children being monitored simultaneously during a week. Further details of each study site and maps of school locations are provided in the appendix (pp 3–9).

Following recruitment, each child was given a bespoke backpack (Dyson, Malmesbury, UK) incorporating a small air pollution monitoring unit with an inbuilt GPS data logger previously used for a study in London, UK.^20^ Before monitoring, each location's research team was provided with the standard operating procedure and training to undertake fieldwork. Children at their respective schools were instructed by the local research team to carry the backpack with them for four full days, including while at school, home, and travelling between the two locations. The unit measured PM_10_, PM_2·5_, and NO_2_, and was placed in the front pocket of the school backpack (appendix p 10). Each child's exposure to NO_2_ and PM, as well as their GPS location, were collected at 1-s intervals, encrypted, and stored in the backpacks' logging unit. All units were calibrated and co-located to a reference monitor before deployment. Due to high uncertainty in performance during calibration, the NO_2_ sensor data were not analysed in this study (appendix p 11). Ethical approval to conduct the study was obtained individually from local research ethics committees for each study site (appendix p 11).

### Procedures

On the first day of monitoring, children filled in a questionnaire in English, with assistance from the field team (appendix pp 12–18). The questionnaire incorporated questions such as self-reported gender, ethnicity, asthma diagnosis, use of an inhaler, and details about their home, including cooker type, lighting, presence of smokers, and other potential air pollution sources. During the monitoring week, children filled out a daily diary (appendix p 19), which recorded any asthma symptoms for the day, the transport modes to get to and from school, and other potential activities that could have resulted in elevated pollution levels.

The GPS coordinate data collected during monitoring were used to determine and tag the microenvironment of each child on a minute-by-minute basis. Activities were split into home, school, and commute microenvironments. Further details on how GPS data were tagged are included in the appendix (p 20). Tagged minute exposure data were used to create summary statistics of pollutant levels for each child in each microenvironment. Data were only analysed if a child collected more than 24 h of continuous valid data within the 96 h monitoring period.

### Statistical analysis

Descriptive statistics are presented by calculating the mean daily exposure for each child in the context of overall exposure, while exposure summarised into microenvironments was averaged across each child's monitoring period. Statistical tests were done with R statistical software (R Core Team, 2018). Due to non-normality, Kruskall Wallis H tests were run to assess mean rank differences between location and microenvironment. Post-hoc tests were run with pairwise Dunn's tests, with the Holm method to adjust p values for multiple comparisons. Linear mixed-effects models were run to identify the most important determinants of children's daily PM_2·5_ exposure. The aim of the model was not to predict exposures but to identify determinants to suggest exposure reduction strategies. Mixed-effects models account for repeated measurements of exposure across participants and locations. Although the main aim of the study was to identify determinants of total daily PM_2·5_ exposure, models were also run to identify determinants of daily PM_2·5_ exposure in each of the three main microenvironments: at home, at school, and commuting. Statistically significant differences were defined as those for which p values were less than 0·05.

The mixed-effects models are presented as follows:

$$\log{\left(daily\;PM_{2.5}\right)}_{ijk}=\alpha +{\left(location/participant\right)}_{ij}+\beta_{n}{\left(fixed\;effect\right)}_{ijk}+...+{ɛ}_{ijk}$$

Where *i* is the index by location, *j* is the index of each child, *k* is the index for the day of monitoring for each child, α represents the fixed mean log exposure for all children, *(location/participant)*_ij_ is the nested random intercept of each child within each location, the βs are the fixed effects for each determinant, and ε_ijk_ is the residual.

Daily average PM_2·5_ exposures were log-transformed and presented as a percent change in daily PM_2·5_ exposure. The model included most variables from a-priori knowledge of factors associated with PM_2·5_ exposure,^11^ such as cooker type, lighting use, commute type, presence of smokers in home, school ground surface, and other potential outdoor sources of PM_2·5_ (waste burning, living near a busy road, and so on). However, several variables were excluded as these models did not result in a low Akaike information criterion (AIC) and were not statistically significant (appendix p 27). The mixed-effects models were run in R statistical software with the lmerTest package.^21^ Multicollinearity between variables was analysed with the variance inflation factor. All variables in the final model produced a variance inflation factor lower than 3. Statistically significant variables were defined as those with p values less than 0·05.

### Role of the funding source

The funder of the study had no role in study design, data collection, data analysis, data interpretation, or writing of the report.

## Results

Of the 330 children who were recruited across all 43 schools, 297 had valid monitoring data, with 1109 days of valid data analysed. Participant data were excluded in instances where no questionnaire was filled out, the child withdrew, GPS tracking revealed that the backpack was not taken to school, or if there was a monitor failure. Characteristics of participants and study sites are summarised in table 1. Children's ethnicity was self-reported as Black apart from three children from Durban who reported their ethnicity as Indian.

**Table 1:** Characteristics of study participants, determinants of exposure recorded, and daily PM_2·5_ exposure summary by location

		**All**	**Blantyre, Malawi**	**Durban, South Africa**	**Harare, Zimbabwe**	**Kumasi, Ghana**	**Lagos, Nigeria**	**Moshi, Tanzania**
Country income status*		**..**	Low income	Upper middle income	Lower middle income	Lower middle income	Lower middle income	Lower middle income
Köppen–Geiger climate classification		**..**	Tropical	Temperate	Temperate	Tropical	Tropical	Temperate
Monitoring period		June–November, 2021	September–November, 2021	August–October, 2021	October–November, 2021	September–November, 2021	June–October, 2021	July–October, 2021
Number of children monitored		297	24 (8%)	47 (16%)	43 (14%)	61 (21%)	61 (21%)	61 (21%)
Number of days monitored†		1109	81 (7%)	180 (16%)	162 (15%)	237 (21%)	221 (20%)	228 (21%)
Near waste burning site								
	Yes	210 (19%)	26 (32%)	17 (9%)	48 (30%)	37 (16%)	22 (10%)	60 (26%)
	No	899 (81%)	55 (68%)	163 (91%)	114 (70%)	200 (84%)	199 (90%)	168 (74%)
Near construction site								
	Yes	94 (8%)	11 (14%)	24 (13%)	2 (1%)	5 (2%)	18 (8%)	34 (15%)
	No	1015 (92%)	70 (86%)	156 (87%)	160 (99%)	232 (98%)	203 (92%)	194 (85%)
Help with cooking								
	Yes	292 (26%)	39 (48%)	9 (5%)	9 (6%)	28 (12%)	73 (33%)	134 (59%)
	No	817 (74%)	42 (52%)	171 (95%)	153 (94%)	209 (88%)	148 (67%)	94 (41%)
Contact with animals								
	Yes	164 (15%)	32 (40%)	20 (11%)	6 (4%)	19 (8%)	21 (10%)	66 (29%)
	No	945 (85%)	49 (60%)	160 (89%)	156 (96%)	218 (92%)	200 (90%)	162 (71%)
Commute mode								
	Motorised	358 (32%)	0	57 (32%)	15 (9%)	145 (61%)	104 (47%)	37 (16%)
	Walk	507 (46%)	64 (79%)	73 (41%)	105 (65%)	64 (27%)	68 (31%)	133 (58%)
	Mixed mode	96 (9%)	7 (9%)	6 (3%)	12 (7%)	22 (9%)	29 (13%)	20 (9%)
	No commute	98 (9%)	9 (11%)	43 (24%)	17 (10%)	5 (2%)	6 (3%)	18 (8%)
	Unclassified	50 (5%)	1 (1%)	1 (1%)	13 (8%)	1 (0%)	14 (6%)	20 (9%)
Cooker location								
	Inside	800 (72%)	38 (47%)	149 (83%)	140 (86%)	158 (67%)	171 (77%)	144 (63%)
	Outside	286 (26%)	43 (53%)	15 (8%)	19 (12%)	79 (33%)	46 (21%)	84 (37%)
	Not reported	23 (2%)	0	16 (9%)	3 (2%)	0	4 (2%)	0
Cooker type								
	Electric	442 (40%)	20 (25%)	136 (76%)	144 (89%)	60 (25%)	78 (35%)	4 (2%)
	Gas	284 (26%)	0	32 (18%)	18 (11%)	83 (35%)	111 (50%)	40 (18%)
	Gas and biomass	240 (22%)	0	8 (4%)	0	66 (28%)	22 (10%)	144 (63%)
	Coal or wood (biomass)	71 (6%)	36 (44%)	0	0	24 (10%)	0	11 (5%)
	Kerosene	23 (2%)	0	0	0	0	2 (1%)	21 (9%)
	Open fire and biomass	41 (4%)	25 (31%)	0	0	4 (2%)	4 (2%)	8 (4%)
	Not reported	8 (1%)	0	4 (2%)	0	0	4 (2%)	0
Lighting use								
	Electric only	365 (33%)	43 (53%)	24 (13%)	16 (10%)	175 (74%)	99 (45%)	8 (4%)
	Candle	198 (18%)	19 (23%)	44 (24%)	85 (52%)	4 (2%)	15 (7%)	31 (14%)
	Candle and other	183 (17%)	3 (4%)	47 (26%)	8 (5%)	15 (6%)	37 (17%)	73 (32%)
	Kerosene lamp and other	92 (8%)	4 (5%)	8 (4%)	0	4 (2%)	10 (5%)	66 (29%)
	Rechargeable, solar lamp or torch	239 (22%)	8 (10%)	33 (18%)	49 (30%)	39 (16%)	60 (27%)	50 (22%)
	Not reported	32 (3%)	4 (5%)	24 (13%)	4 (2%)	0	0	0
Smoking at home								
	No smokers	880 (79%)	63 (78%)	111 (62%)	108 (67%)	222 (94%)	167 (76%)	209 (92%)
	Presence of smokers	211 (19%)	18 (22%)	62 (34%)	54 (33%)	15 (6%)	43 (19%)	19 (8%)
	Not reported	18 (2%)	0	7 (4%)	0	0	11 (5%)	0
Self-reported gender								
	Female	663 (60%)	50 (62%)	123 (68%)	76 (47%)	155 (65%)	138 (62%)	121 (53%)
	Male	446 (40%)	31 (38%)	57 (32%)	86 (53%)	82 (35%)	83 (38%)	107 (47%)
Live near a busy road								
	Yes	808 (73%)	35 (43%)	120 (67%)	113 (70%)	171 (72%)	170 (77%)	199 (87%)
	No	278 (25%)	46 (57%)	44 (24%)	49 (30%)	66 (28%)	44 (20%)	29 (13%)
	Not reported	23 (2%)	0	16 (9%)	0	0	7 (3%)	0
School ground surface								
	Loose dirt	648 (58%)	81 (100%)	0	0	163 (69%)	176 (80%)	228 (100%)
	Packed dirt	65 (6%)	0	0	0	20 (8%)	45 (20%)	0
	Broken paving	74 (7%)	0	74 (41%)	0	0	0	0
	Paved	322 (29%)	0	106 (59%)	162 (100%)	54 (23%)	0	0
Day of week								
	Monday	288 (26%)	21 (26%)	47 (26%)	42 (26%)	61 (26%)	61 (28%)	56 (25%)
	Tuesday	279 (25%)	21 (26%)	45 (25%)	42 (26%)	60 (25%)	51 (23%)	60 (26%)
	Wednesday	274 (25%)	19 (23%)	45 (25%)	39 (24%)	58 (24%)	56 (25%)	57 (25%)
	Thursday	268 (24%)	20 (25%)	43 (24%)	39 (24%)	58 (24%)	53 (24%)	55 (24%)
Daily mean air temperature (SD), °C‡		24·2 (4·2)	28·2 (4·2)	19·1 (1·9)	22·5 (4·8)	27·4 (2·0)	27·0 (1·2)	22·5 (2·2)
Daily mean relative humidity (SD)‡		67·7% (16·1)	44·4% (14·2)	72·1% (8·9)	54·8% (18·1)	77·1% (8·9)	82·2% (5·7)	58·9% (8·9)
Daily mean wind speed, ms^−1^‡		3·1 (1·3)	3·8 (1·3)	2·8 (0·8)	4·0 (1·2)	1·8 (0·6)	3·1 (0·8)	3·7 (1·5)
Children's personal daily mean PM_2·5_ exposure, μg/m^3^								
	Median (range)	22·9 (3·7–213·9)	41·8 (18·2–213·9)	16·0 (4·4–197·7)	22·1 (5·9–87·9)	17·9 (5·5–79·2)	23·0 (3·7–78·8)	27·4 (8·8–131·9)
	Arithmetic mean (SD)	28·0 (20·3)	52·4 (35·5)	26·7 (27·2)	24·1 (12·7)	20·5 (11·3)	26·4 (11·5)	32·2 (17·3)
	Geometric mean (SD)	23·4 (1·8)	45·7 (1·6)	18·8 (2·2)	21·4 (1·6)	18·3 (1·6)	24·1 (1·5)	29·1 (1·5)

Data are n (%), mean (SD), or median (range). PM_2·5_=particulate matter <2·5 μm in diameter.

* As designated by the World Bank.^23^

† Denominators of the percentages reported in this table.

‡ Data collected from worldmet.^24^

Overall median daily PM_2·5_ exposure was 22·9 μg/m^3^ and varied drastically between children and locations (range 3·7–213·9 μg/m^3^; table 1). Only 227 (20%) of 1109 days monitored were lower than the current WHO 24 h PM_2·5_ exposure health guideline^22^ of 15 μg/m^3^. Personal PM_10_ exposure largely followed a similar trend to PM_2·5_, with a median daily exposure of 33·0 μg/m^3^ (range 6·2–363·6 μg/m^3^) across all six locations (appendix p 29). However, 1020 (92%) of 1109 days monitored were lower than the current WHO 24 h PM_10_ exposure health guideline of 45 μg/m^3^.

There was a significant difference in personal PM_2·5_ exposure between children at different locations (p<0·0001). Children's exposure in Blantyre (median 41·8 μg/m^3^) was significantly higher than in all other locations, whereas children's exposure in Durban (16·0 μg/m^3^) was significantly lower than in all locations except for Kumasi (17·9 μg/m^3^; p=0·27; table 1).

**Figure 1:**
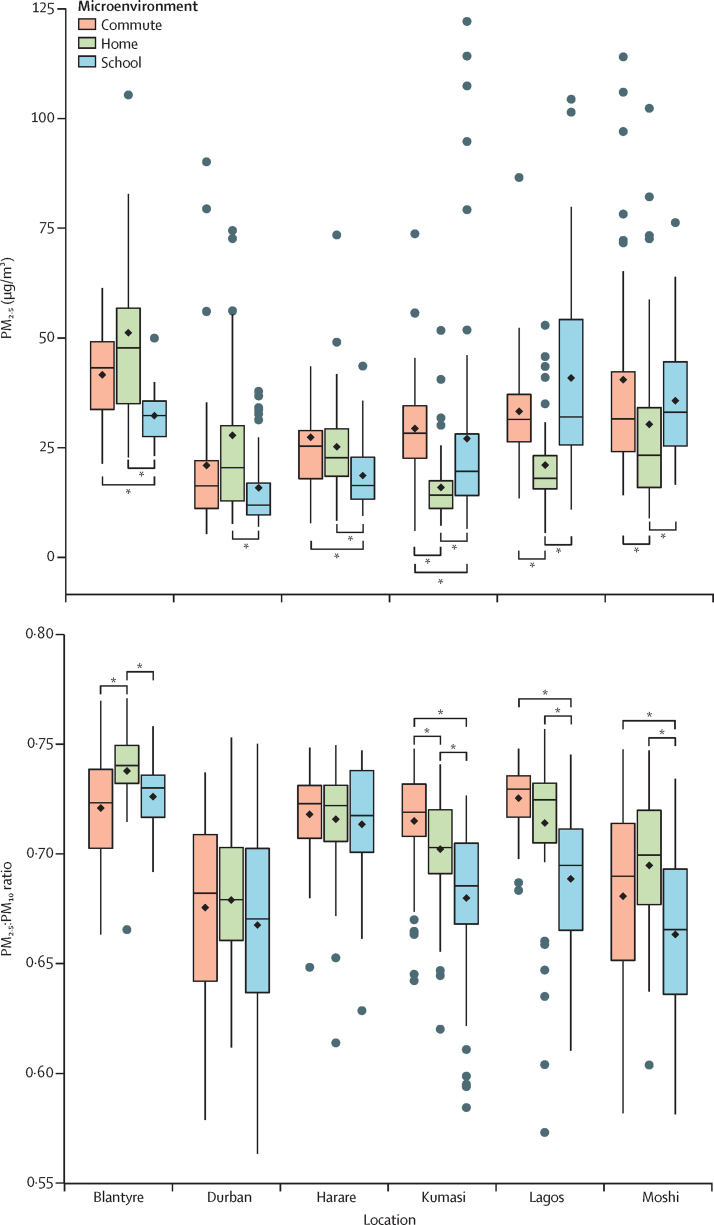
School children's mean PM2.5 exposure and PM2.5:PM10 ratio in different microenvironments across six locations in sub-Saharan Africa (A) School children's mean personal PM_2.5_ exposure measured in commute, home, and school microenvironments during 4 days (96 h) of monitoring across six locations in sub-Saharan Africa. (B) School children's mean personal PM_2.5_:PM_10_ ratio measured in commute, home, and school microenvironments for 4 days (96 h) of monitoring across six locations in sub-Saharan Africa. In both panels, the horizontal black lines denote the median proportion; boxes depict the IQR; vertical lines indicate 1·5 times the IQR, and dots represent proportions outside the range of these values. Arithmetic means are represented by black diamonds. Statistically significant differences are according to the Dunn's test. PM=particulate matter. *p<0·05.

Personal PM_2·5_ exposure was summarised by microenvironment for each child. Locations fell broadly into two patterns of exposure: in Blantyre, Durban, and Harare, children had significantly higher exposures at home (median 48·0 μg/m^3^ in Blantyre, 20·9 μg/m^3^ in Durban, and 22·7 μg/m^3^ in Harare) and while commuting (43·2 μg/m^3^ in Blantyre, 16·3 μg/m^3^ in Durban, and 25·4 μg/m^3^ in Harare) than at school (32·3 μg/m^3^ in Blantyre, 11·9 μg/m^3^ in Durban, and 16·4 μg/m^3^ in Harare; figure 1A, appendix p 31). By contrast, in Kumasi, Lagos, and Moshi, children had significantly higher exposures at school (median 19·6 μg/m^3^ in Kumasi, 32·0 μg/m^3^ in Lagos, and 33·1 μg/m^3^ in Moshi) than at home (14·2 μg/m^3^ in Kumasi, 18·0 μg/m^3^ in Lagos, and 23·6 μg/m^3^ in Moshi). Only children in Kumasi had significantly higher exposures while commuting (28·3 μg/m^3^) than at school and at home.

PM_2·5_:PM_10_ ratios were also compared between microenvironments. PM_2·5_:PM_10_ ratios were significantly lower at school compared with at home in Moshi, Lagos, Kumasi, and Blantyre, whereas there was no significant difference between any microenvironment ratios in Harare and Durban (figure 1B, appendix p 32). The PM_2·5_:PM_10_ ratio while commuting was significantly higher than at home in Kumasi but lower than PM_2·5_:PM_10_ ratios at home in Blantyre. There was no significant difference in PM_2·5_:PM_10_ ratios between commute and home environments in Lagos and Moshi.

**Figure 2:**
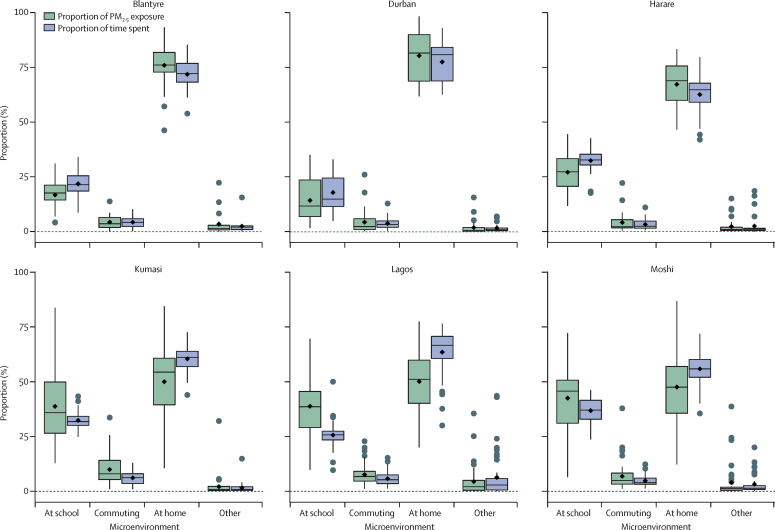
Proportion of PM_2·5_ exposure and time spent split between different locations The horizontal black lines denote the median proportion; boxes depict the IQR; vertical lines indicate 1·5 times the IQR, and grey dots represent proportions outside the range of these values. Mean proportions are represented by black diamonds. PM=particulate matter.

The proportion of total PM_2·5_ exposure and proportion of time spent in each microenvironment was also analysed to identify the most influential microenvironments on total exposure (figure 2, appendix p 32). A further 32 children were removed from this analysis because these children had more than 12 h of data with no GPS signal, thus preventing location tagging.

The largest proportion of time spent at home and the shortest time spent in school was in Blantyre (median at home: 72%; median at school: 21%) and Durban (median at home: 81%; median at school: 15%), reflecting the impact of the COVID-19 pandemic on school hours in these cities.

The proportion of total PM_2·5_ exposure reinforced the findings of the microenvironment exposure analysis, with children in Blantyre (median 76%), Durban (median 82%), and Harare (median 69%) having more than 65% of their total exposure in the home environment, while children in Kumasi (36%), Lagos (39%), and Moshi (46%) had more than 35% of their exposure in the school environment (figure 2). Commuting exposures only contributed 2–8% of total exposure due to the short amount of time spent (2–6%) in this microenvironment.

**Figure 3:**
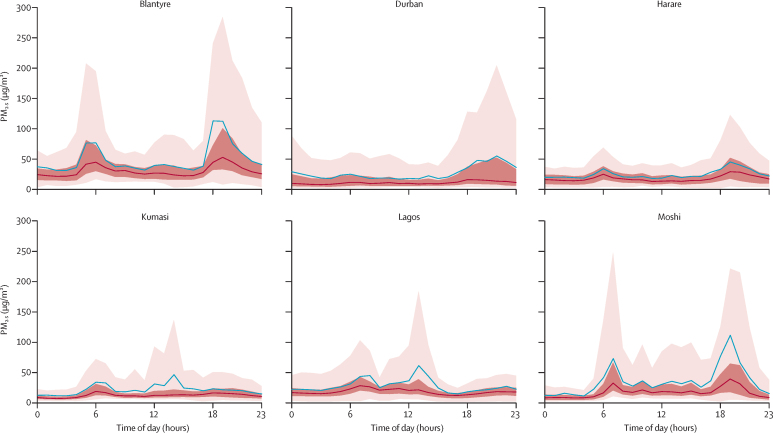
Diurnal hourly PM_2.5_ exposure for all children by location The red line represents median exposure, the blue line represents mean exposure, the dark shaded areas are the IQR of the exposure values, and the light shaded areas are the 5th to 95th percentile of exposure values. PM=particulate matter.

Diurnal exposure also varied significantly between locations (figure 3), with the peaks in diurnal exposure observed coinciding with commuting or cooking at home. The morning peaks in Moshi, Kumasi, Lagos, and Harare were predominantly observed due to commuting exposures. The evening peaks in exposure in Moshi, Durban, and Harare were likely to be due to cooking in the home. For Blantyre, there were two noticeable peaks in PM_2·5_ exposure that were higher than 50 μg/m^3^ at around 0500–0600 h and 1800–1900 h, which were both suggested to be due to cooking in the home. Lagos was the only location where a peak in exposure occurred during school time (1300 h). For Moshi, which also had comparatively high school exposures, the diurnal trends highlighted that these exposures were relatively persistent throughout the day rather than specific high-exposure events.

The mixed-effects model highlighted important determinants of daily PM_2·5_ exposure for children (table 2). On days when children did not commute (this also included children whose commute to school was less than 5 min), they had, on average, an 18·3% (95% CI 8·4–26·8) lower daily PM_2·5_ exposure compared with days on which they walked to and from school. However, there was no significant difference in daily exposure for children who walked to and from school compared with those who commuted with motorised transport.

**Table 2:** Fixed-effects determinants from mixed-effects model for children's logged daily personal PM_2.5_ exposure

		**Reference group**	**Percentage change**	**95% CI**	**p value**
Near waste burning site		No	0·4%	−6·8 to 8·7	0·91
Near construction site		No	4·5%	−6·8 to 17·3	0·46
Help with cooking		No	2·0%	−5·8 to 10·8	0·64
Contact with animals		No	1·9%	−6·8 to 11·6	0·69
Commute type					
	Motorised	Walk	2·7%	−6·1 to 11·8	0·56
	Mixed mode	Walk	0·7%	−10·5 to 12·3	0·91
	No commute	Walk	−18·3%	−26·8 to −8·4	0·0005
	Not recorded	Walk	−13·1%	−25·7 to 1·5	0·086
Cooker location					
	Outside	Inside	−14·5%	−22·4 to −5·8	0·0027
	Not reported	Inside	0·0	−33·7 to 19·5	0·46
Cooker type					
	Gas	Electric	13·4%	1·2 to 26·8	0·037
	Gas and biomass	Electric	8·0%	−6·0 to 24·4	0·30
	Coal or wood (biomass)	Electric	27·1%	3·9 to 56·3	0·028
	Kerosene	Electric	15·5%	−13·2 to 54·1	0·34
	Open fire and biomass	Electric	28·8%	1·1 to 64·5	0·049
	Not reported	Electric	54·9%	−4·5 to 150·0	0·089
Lighting use					
	Candle	Electric only	14·0%	−0·2 to 30·5	0·067
	Candle and mixed	Electric only	10·2%	−3·7 to 26·4	0·18
	Kerosene lamp and mixed	Electric only	30·2%	9·1 to 55·2	0·0052
	Rechargeable, solar lamp or torch	Electric only	18·1%	4·9 to 33·1	0·0090
	Not reported	Electric only	1·7%	−22·9 to 34·6	0·91
Smoking at home					
	Presence of smokers	No smokers	23·0%	10·8 to 36·4	0·0002
	Not reported	No smokers	9·2%	−21·3 to 50·9	0·61
Gender					
	Male	Female	5·7%	−2·4 to 14·7	0·20
Lives near busy road					
	No	Yes	0·9%	−8·0 to 11·0	0·85
	Not reported	Yes	11·8%	−17·4 to 52·4	0·49
School ground surface					
	Packed dirt	Loose dirt	−6·6%	−21·5 to 11·1	0·46
	Broken paving	Loose dirt	8·6%	−18·0 to 45·0	0·59
	Paved	Loose dirt	−37·2%	−48·2 to −22·9	<0·0001
Day of the week					
	Tuesday	Monday	2·6%	−4·6 to 10·5	0·50
	Wednesday	Monday	3·7%	−3·6 to 11·9	0·34
	Thursday	Monday	−0·3%	−7·7 to 8·1	0·94
Mean daily air temperature (°C)		..	5·6%	3·5 to 7·5	<0·0001
Mean daily relative humidity (%)		..	0·2%	−0·3 to 0·6	0·35
Mean daily wind speed (ms^−1^)		..	−8·4%	−11·1 to −5·6	<0·0001

Percentage change calculated as follows: (exp(effect estimate) – 1) × 100%. PM_2.5_=particulate matter <2·5 μm in diameter.

Children who lived in a home with an outdoor cooker had, on average, a 14·5% (95% CI 5·8–22·4) lower daily PM_2·5_ exposure than those who lived in a home with an indoor cooker. However, the cooker location was not a significant determinant in the mixed-effects model that included only at-home PM_2·5_ exposure (appendix pp 40–41). Children had, on average, a higher daily PM_2·5_ exposure if they lived in a home in which the main cooker was gas (13·4%; 95% CI 1·2–26·8), coal or wood (27·1%; 3·9–56·3), or open fire (28·8%; 1·1–64·5) than if they lived in a home in which the main cooker was electric.

Children who used kerosene lamps (30·2%; 95% CI 9·1–55·2) and rechargeable lamps, solar lamps, and torches (18·1%; 4·9–33·1) as an alternative lighting source had, on average, significantly higher daily exposures than those children who only used electric lighting. The at-home model only found a weak difference between children who used kerosene lamps and electric lighting, while no difference in exposure was observed for those children who used rechargeable lamps, solar lamps, and torches (appendix pp 40–41). Children who lived in a home with a smoker had, on average, 23·0% (95% CI 10·8–36·4) higher daily average PM_2·5_ exposure than those who did not.

The type of school ground also appeared to influence exposure, with children who went to schools with paved grounds having, on average, a 37·2% (95% CI 22·9–48·2) lower daily average PM_2·5_ exposure than children who went to schools with grounds that were covered with loose dirt.

In terms of meteorological values, there was, on average, a 5·6% (95% CI 3·5–7·5) higher daily PM_2·5_ exposure for every 1°C increase in daily mean temperature, while there was an 8·4% (5·6–11·1) lower exposure for every ms^–1^ increase in average daily wind speed. Changes in average daily relative humidity did not result in any significant difference in exposure.

Several determinants did not relate to significant differences in daily exposure, including whether a child was living near a waste burning site or construction site or near a busy road. The self-reported gender of the child and day of the week were not significantly associated with daily exposure. However, there were differences in exposure for these determinants in the mixed-effects model that included only at-school PM_2·5_ exposure (appendix p 40, 42), with significantly higher exposures observed when children were near a waste burning site, lower exposures when they were near a construction site, and higher exposures for males compared with females.

## Discussion

This study conducted personal PM exposure measurements for 297 school children with asthma in six urban cities, resulting in 1109 days of data collected. To the best of our knowledge, this is the largest high-resolution personal PM_2·5_ exposure monitoring study conducted in cities in sub-Saharan Africa to date and the first to measure exposures in children with asthma in Africa. By using a novel GPS algorithm to determine participants' locations, the analysis also allowed for direct comparison of exposure levels and sources, and highlighted the importance of different microenvironments. Overall, PM_2·5_ exposures measured were inverse to relative country income status,^23^ with children in the lowest-income country (Blantyre, Malawi) reporting the highest exposures while children in the highest-income countries (Durban, South Africa, and Kumasi, Ghana) reported the lowest exposures. Only 20% of days monitored were lower than the daily WHO PM_2·5_ guideline of 15 μg/m^3^. On average, the exposures measured in this study were similar to those observed in studies conducted in urban areas in sub-Saharan Africa,^13^, ^14^, ^15^, ^16^ but lower than those conducted in rural sub-Saharan Africa.^11^, ^25^

Microenvironment exposure across the six cities varied, with the highest exposures observed at school (Lagos and Moshi), at home (Durban, Harare, and Blantyre), or while commuting (Kumasi), highlighting the importance of context in diverse urban study locations. This is in contrast to exposure studies completed in rural areas, which focus on household air pollution based on the assumption that this is the highest exposed microenvironment.^11^ However, it is also important to note that due to the time spent in the home environment, the proportion of daily PM_2·5_ exposure was the highest in this microenvironment for all six cities.

Lower PM_2·5_:PM_10_ ratios in the school environment in Blantyre, Kumasi, Lagos, and Moshi suggested that the source of pollution is likely to be from natural sources such as resuspended dust from school grounds, while higher ratios while commuting and at home are likely to be from anthropogenic sources.^17^, ^18^ Children in Durban and Harare went to schools that had paved grounds and therefore there was no difference in PM_2·5_:PM_10_ ratios between microenvironments in these cities. Although there has been debate around whether natural sources of PM have less adverse health effects than anthropogenic sources, a recent review on desert dust^26^ suggested links between this pollutant and asthma exacerbations. This suggests that PM exposure from both anthropogenic and dust sources should be reduced to improve health outcomes.

The development of a mixed-effects model allowed assessment of determinants controlling for confounding variables, an essential method to correctly interpret personal exposures across multiple locations and microenvironments. The most important determinants were the presence of smokers in the home, use of biomass or open fire for cooking, and kerosene lamps for lighting. A surprising result was the higher exposures observed for children who used rechargeable lamps, solar lamp, or torches. Frequent use of this type of lighting suggests a high prevalence of power cuts, which might have resulted in the use of diesel generators, which could have increased PM_2·5_ exposure.^27^ These results suggest that increasing the availability and reliability of electricity, encouraging the use of electricity in the home for cooking and lighting, and reducing smoking rates, would decrease PM_2·5_ exposure.

For school determinants, we found a clear exposure differential between paved school surfaces and those with loose dirt. This suggests that a potential way to reduce PM exposure at school would be to pave school grounds^13^ or dampen the resuspension of loose dirt. Furthermore, at-school exposures in this study were two to six times higher than exposures reported in children in a similar study done in London, UK, where exposures were 5·4 μg/m^3^.^20^

In contrast to high-income countries, we found that commuting did not make up a significant proportion of daily exposure,^28^ and walking compared to taking motorised transport did not result in lower PM_2·5_ exposure.^29^ This observation might be due to the prevalence of unpaved roads along commuting routes or the fact that commuting only contributed a small amount to total daily exposure. Another finding was the insignificant difference in exposure for children who lived close to busy roads; this finding could potentially be explained by a larger influence of non-vehicular sources of PM_2·5_ in this region, such as resuspended dust and biomass cooking.

The determinants identified were generally supported by the small amount of published literature on personal exposure in African cities. Arku and colleagues^13^ found similar results to ours, with higher PM_2·5_ exposures in homes that used biomass compared with non-biomass and for children whose school grounds had loose dirt compared with those whose school grounds were paved. However, a key difference was that their study found higher exposures in female students compared with male students. There have been conflicting results in relation to differences in exposures between genders, with some studies finding no difference in exposure between males and females,^11^, ^25^ while our at-school model found a higher exposures for males than for females. Another study in peri-urban Accra, Ghana, found higher PM_2·5_ exposures for adults who used wood burning for cooking compared with liquified petroleum gas or coal.^14^ A study in Dar es Salaam, Tanzania, found lower PM_2·5_ exposures for pregnant women who cooked outdoors compared with those who cooked indoors, but there was no difference in exposure with the use of different cooking fuels.^15^ A systematic review on smoking and non-smoking households in high-income countries found higher concentrations of PM_2·5_ in homes with a smoker than in those without,^30^ but few studies have been conducted on smoking exposures in Africa.^16^

This study had some limitations. Despite collecting one of the largest datasets of personal exposure measurements in urban centres in Africa, due to the sample size the measurements are unlikely to be fully representative of all children across each city and the exposures measured do not provide insight into seasonal variations in exposure. Furthermore, because monitoring was conducted during the COVID-19 pandemic, some of the exposures might not be representative of typical daily exposure. It was also difficult to disentangle the potential effects of household income in identifying the determinants of exposure, as children who did not have continuous electricity and attended schools that do not have paved grounds are typically likely to be living in lower socioeconomic areas. The addition of outdoor ambient monitoring would have also assisted with the identification of other potential outdoor sources of PM_2·5_ around the home and school, and might have better highlighted the personal determinants of exposure. The health effects of personal PM_2·5_ exposure were not ascertained in this analysis, since the aim of this study was to characterise exposures and identify exposure reduction strategies. However, some children in the present study also undertook repeated lung function measurements, and the analysis of these data is in progress. These results will provide further insight into the effect of PM exposure on asthma symptoms in sub-Saharan Africa.

In summary, this study is, to our knowledge, one of the most comprehensive analyses to provide evidence for targeted interventions within personal microenvironments for the reduction of PM exposure in school children with asthma symptoms in urban centres in sub-Saharan Africa. Although generic policies to reduce PM exposure in all locations could be implemented, the benefit of using high time-resolution monitors and GPS highlighted that the most efficient way to reduce daily exposure in Harare, Durban, and Blantyre would be to focus on the home environment, while in Kumasi, Lagos, and Moshi the focus should be on the school environment. Our findings suggest that the most effective changes would be to provide paving in school grounds, increase the use of clean fuels for cooking and lighting in homes, and discourage smoking within homes. The results of this study can be used to inform future personal exposure assessments and interventions for children living in urban centres in Africa.

## Data sharing

Due to the collection of sensitive data in this study, including participant GPS locations, data are not available to be shared to protect participant confidentiality.

## Declaration of interests

JG reports grants from OM Pharma, Mariomed, and Biotech; advisory board membership on OM Pharma, GlaxoSmithKline, and AstraZeneca; payment from Hodge Jones & Allen Solicitors for medical advice to a coroner's inquest into a child who died from asthma. All other authors declare no competing interests.
