# Supplementary material for: Characterising sources of PM2·5 exposure for school children with asthma: a personal exposure study across six cities in sub-Saharan Africa
Source: Lancet Child Adolesc Health. 2024 Jan;8(1):17–27. doi: 10.1016/S2352-4642(23)00261-4 (PMC10716619; doi:10.1016/S2352-4642(23)00261-4)
Supplement: Supplementary appendix [file mmc1.pdf]

THE LANCET
Child & Adolescent Health

Supplementary appendix

This appendix formed part of the original submission and has been peer reviewed. We post it as supplied by the authors.

Supplement to: Lim S, Said B, Zurba L, et al. Characterising sources of PM2·5 exposure for school children with asthma: a personal exposure study across six cities in sub-Saharan Africa. *Lancet Child Adolesc Health* 2023; published online Nov 21. https://doi.org/10.1016/S2352-4642(23)00261-4.

Supplementary information

Characterising sources of $PM_{2.5}$ exposure for school children with asthma in six Sub-Saharan African cities

Shanon Lim[1,2*], Bibie Said[9,11], Lindsay Zurba[4], Gioia Mosler[3], Emmanuel Addo-Yobo[16], Olayinka Olufunke Adeyeye[8], Bernard Arhin[17], Dimitris Evangelopoulos[1], Victoria Temitope Fapohunda[8], Farida Fortune[12], Chris J. Griffiths[14], Sbekezelo Hlophe[6], Marian Kasekete[7], Scott Lowther[18], Refiloe Masekela[6], Elizabeth Mkutumula[13], Blandina Theophil Mmbaga[9,10], Hilda Angela Mujuru[7], Rebecca Nantanda[15], Lovemore Mzati Nkhalamba[13], James S. Ngocho[9,10], Oluwafemi Tunde Ojo[8], Sandra Kwarteng Owusu[17], Sunshine Shaibu[8], Ismail Ticklay[7], Jonathan Grigg[3+], Benjamin Barratt[1,5+]

[+]Indicates equal contribution as Principal Investigators

[1]MRC Centre for Environment and Health, Environmental Research Group, Imperial College London, SW7 2AZ, London, UK
[2]Department of Civil and Environmental Engineering, Faculty of Engineering, The University of Auckland, Auckland 1142, New Zealand
[3]Centre for Genomics and Child Health, Blizard Institute, Barts and The London Faculty of Medicine and Dentistry, Queen Mary University of London, London, UK
[4]Education for Health Africa, Durban, South Africa
[5]NIHR NPRU in Environmental Exposures and Health, Imperial College London, SW7 2AZ, London, UK
[6]Department of Paediatrics and Child Health, Nelson R Mandela School of Clinical Medicine, College of Health Sciences, University of KwaZulu Natal, Durban, South Africa
[7]University of Zimbabwe Faculty of Medicine and Health Sciences, Harare, Zimbabwe
[8]Department of Medicine, Lagos State University College of Medicine, and Lagos State University Teaching Hospital, Ikeja Lagos, Nigeria
[9]Kilimanjaro Clinical Research Institute, Kilimanjaro Christian Medical Centre, Moshi, Tanzania
[10]Kilimanjaro Christian Medical University College, Moshi, Tanzania
[11]Kibong'oto Infectious Disease Hospital, Hai, Tanzania
[12]Centre for Oral immunobiology and Regenerative Medicine, Faculty of Medicine and Dentistry, Queen Mary University of London, London, UK
[13] Malawi Liverpool Wellcome Programme, Blantyre, Malawi
[14] Asthma UK Centre for Applied Research, Wolfson Institute of Population Health, Faculty of Medicine and Dentistry, Queen Mary University of London, London, UK
[15]Makerere University Lung Institute, Makerere College of Health Sciences, Kampala Uganda
[16]Department of Child Health- School of Medicine and Dentistry, Kwame Nkrumah University of Science and Technology, Kumasi, Ghana
[17]Komfo Anokye Teaching Hospital, Kumasi, Ghana
[18]Dyson Technology Limited, Malmesbury, Wiltshire, United Kingdom

Contents

1. Description of locations, participants and picture of monitor 3
2. Monitor co-location information 11
3. Ethical information 11
4. Example of before monitoring questionnaire 12
5. Example of participant daily diary template 19
6. Identifying participant microenvironments using GPS coordinates 20
7. Mixed effects models variables 27
8. Overall exposure summary 28
9. Microenvironment exposure results 31
10. Descriptive statistics on potential determinants of personal exposure 33
11. Additional mixed effects models for sensitivity analysis 40
12. References 44

1. Description of locations, participants and picture of monitor

Blantyre, Malawi: The children were recruited from thirteen schools in Blantyre (Figure S1.1), with one to four children recruited from each school. Most children in Blantyre reside in brick and mud houses. The roofs of these houses are typically iron sheets while a few are thatched with grass. Most of these houses have cement floors with a few that have mud floors. Charcoal and wood are the most commonly used fuels for cooking, with a few households using electricity. Cooking is conducted indoors (kitchen), at the veranda (khonde) and open air. The school grounds are generally unpaved, however, they usually sprinkle water to minimize dust. Most classrooms in Blantyre are well ventilated and classes are conducted indoors. The children recruited in this study were typically from low to medium income families. Children in Blantyre were affected by the COVID pandemic with reduced school hours to reduce virus transmission.

Durban, South Africa: The children were recruited from eight schools in Durban (Figure S1.2), with three to eight children recruited from each school. The children were typically from low-medium income families. They predominantly resided in brick houses and most had electricity, however, frequent power cuts mean that alternative sources of power are often required. Cooking is commonly conducted indoors. Participants in Durban were significantly impacted by the COVID pandemic compared to other countries. Typically, children would go to school each day from Monday to Friday, however due to restrictions, some of the schools only allowed children to go to school on alternate days to reduce potential virus transmission during the monitoring period. School grounds are generally paved in Durban.

Harare, Zimbabwe: The children were recruited from four schools in the west of Harare (Figure S1.3), with two to twenty five children recruited from each school. The participants were typically from low-income families. They predominantly resided in brick houses and most had electricity, however, frequent power cuts mean that alternative sources of power are often required. Cooking is commonly conducted indoors. School grounds were paved.

Kumasi, Ghana: The children were recruited from seven schools in Kumasi (Figure S1.4), with five to twenty one children recruited from each school. School grounds are typically unpaved however there were two schools which were paved. Kitchen areas in Kumasi are typically open air and detached from the main house. The participants were from mixed income from low to high income families.

Lagos, Nigeria: The children were recruited from six schools in Ikeja, Lagos (Figure S1.5), with six to fourteen children recruited from each school. All of the schools have unpaved, dusty grounds with enclosed classrooms. The participants were typically from low-medium income families. They predominantly resided in brick houses and most had electricity, however, frequent power cuts mean that alternative sources of power are often required. Cooking is commonly conducted indoors.

Moshi, Tanzania: The children were recruited from five schools in Moshi Municipal (Figure S1.6), with six to eighteen children recruited from each school. All schools have unpaved grounds filled with laterite. The participants were typically from middle to low-income families. They predominantly live in brick houses and most have electricity, however, in case of power cuts alternative sources are used.

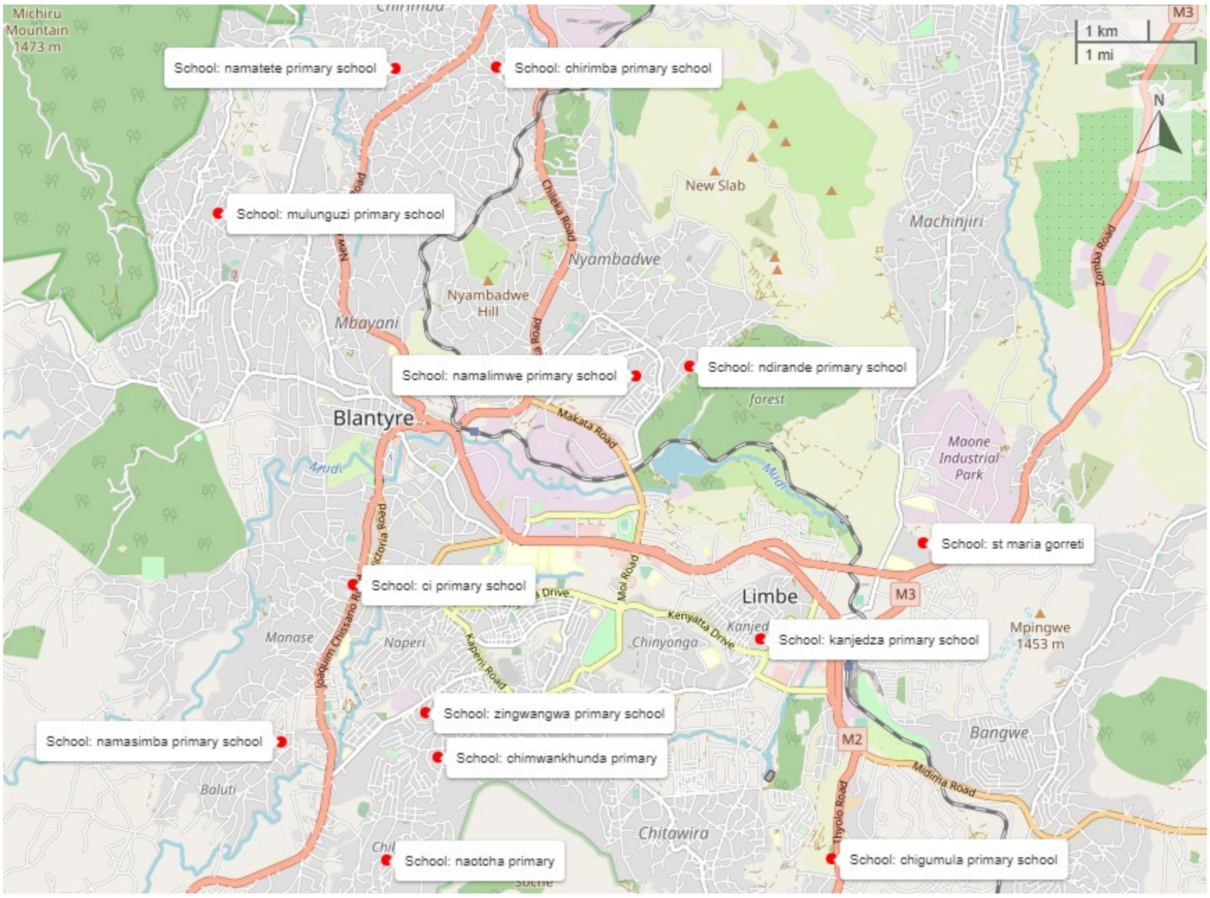

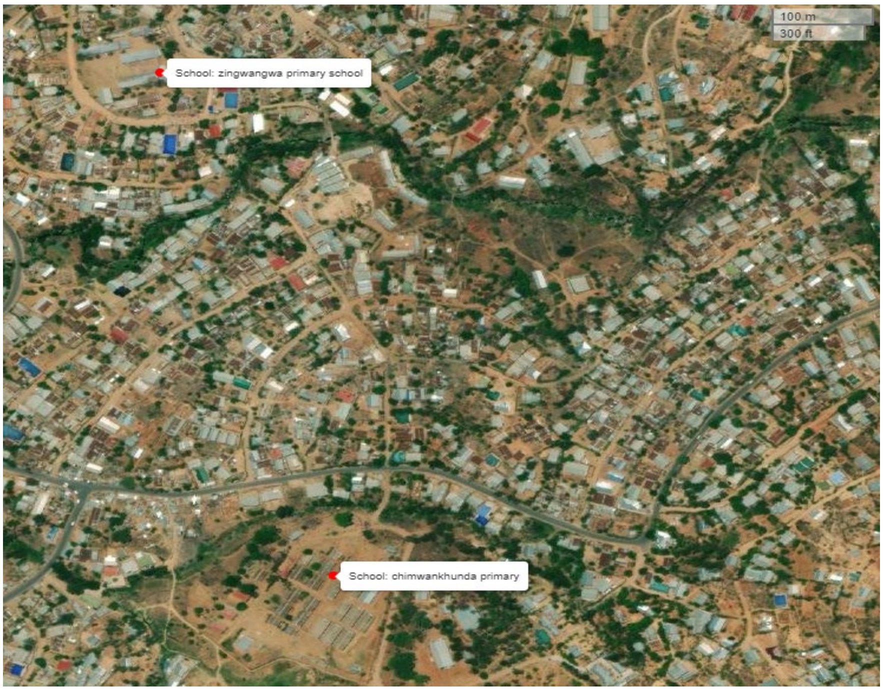

Figure S1.1: Location of thirteen schools with participating students in Blantyre, Malawi and satellite image of an example of typical school grounds in Blantyre, showing Zingwangwa and Chimwankhunda Primary School.

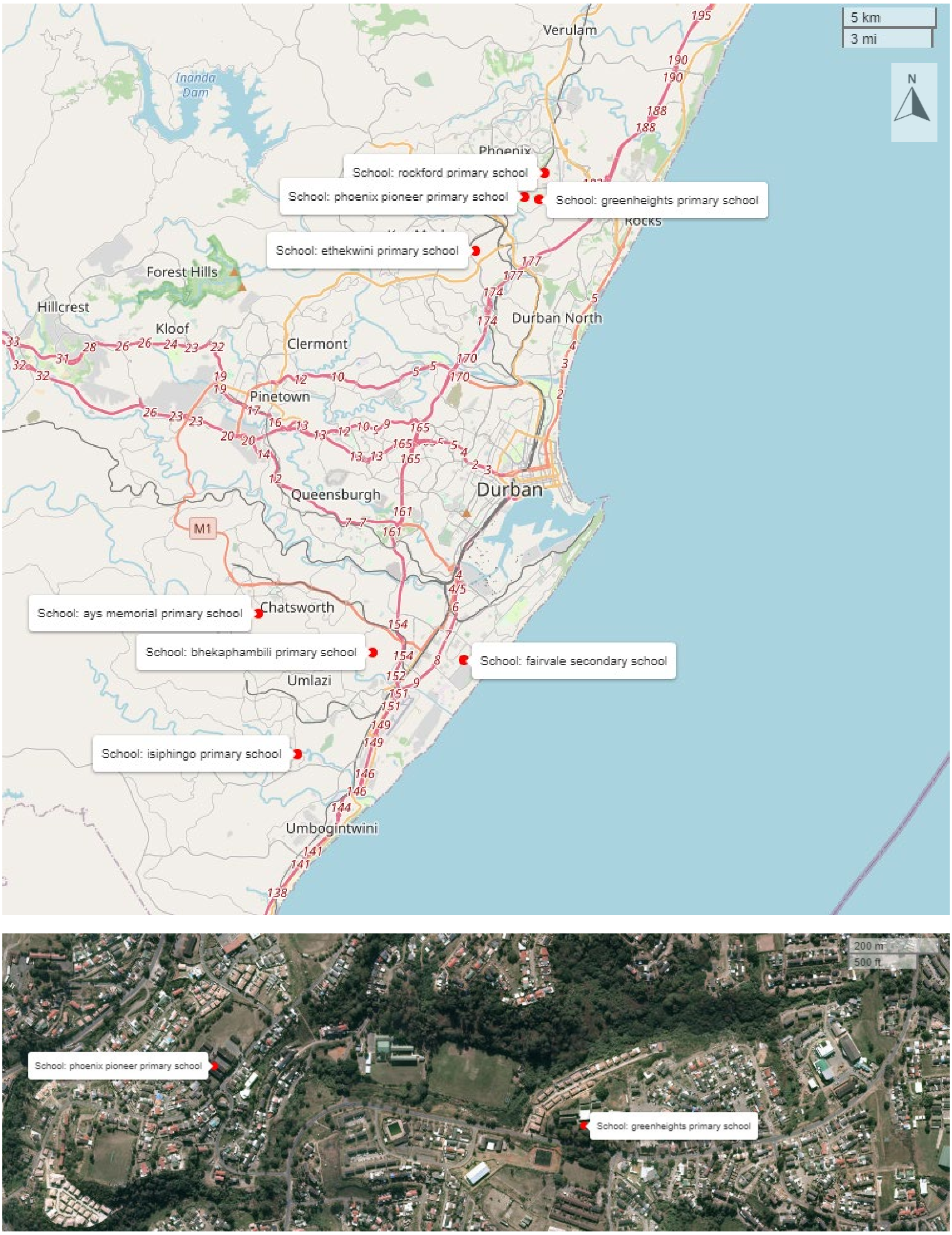

Figure S1.2: Location of eight schools with participating students in Durban, South Africa and satellite image of an example of typical school grounds in Durban, showing Phoenix Pioneer and Greenheights Primary School.

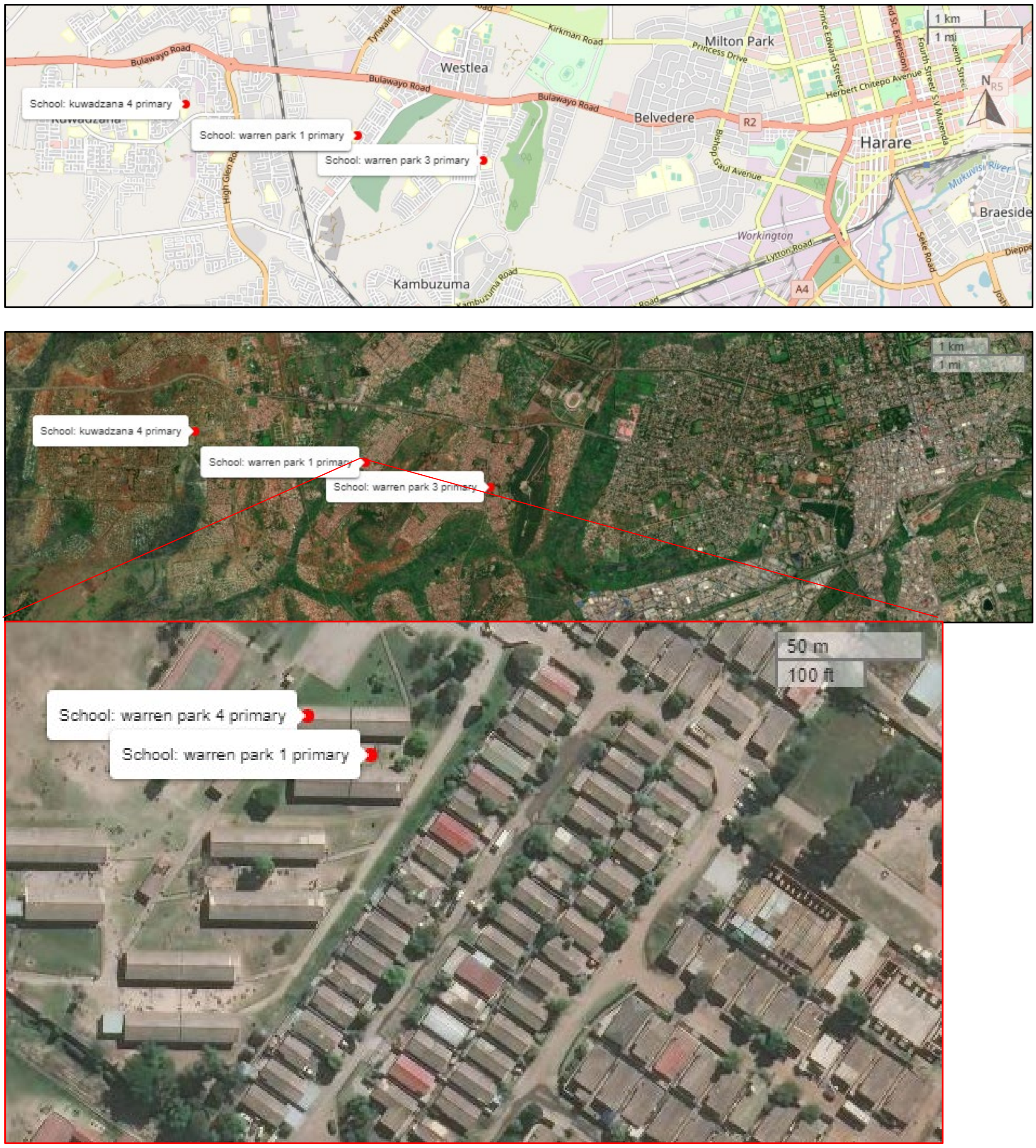

Figure S1.3: Location of four schools with participating students in Harare, Zimbabwe and satellite image of an example of typical school grounds in Harare.

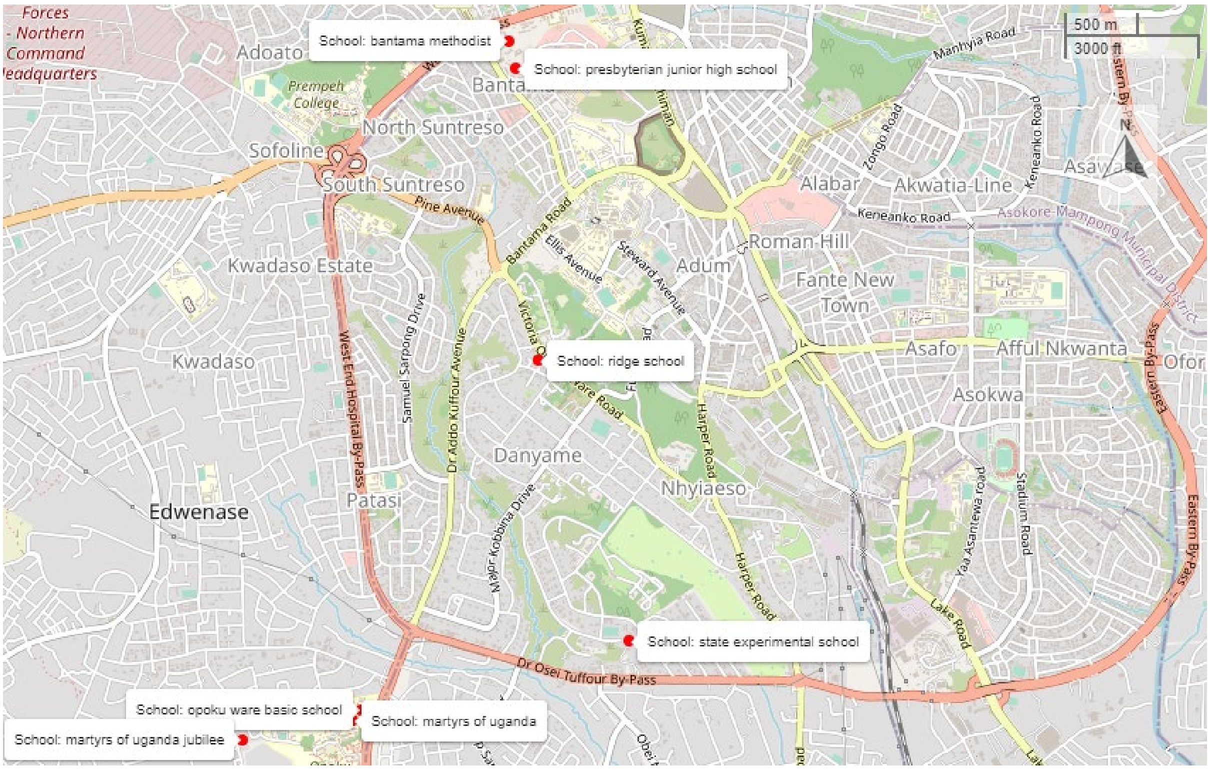

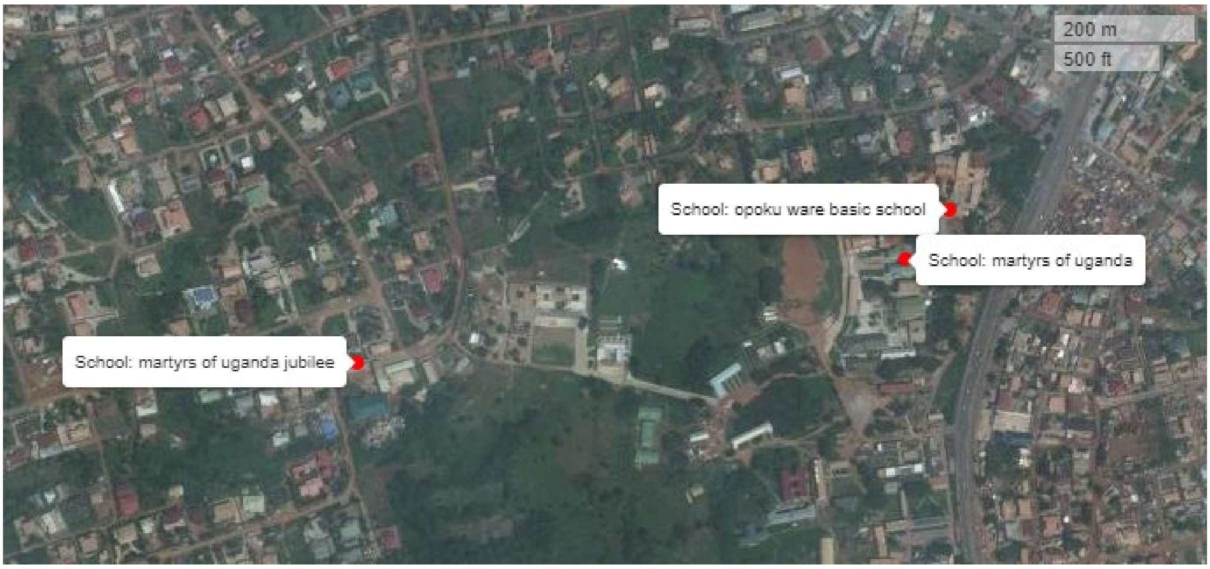

Figure S1.4: Location of seven schools with participating students in Kumasi, Ghana and satellite image of an example of typical school grounds in Kumasi.

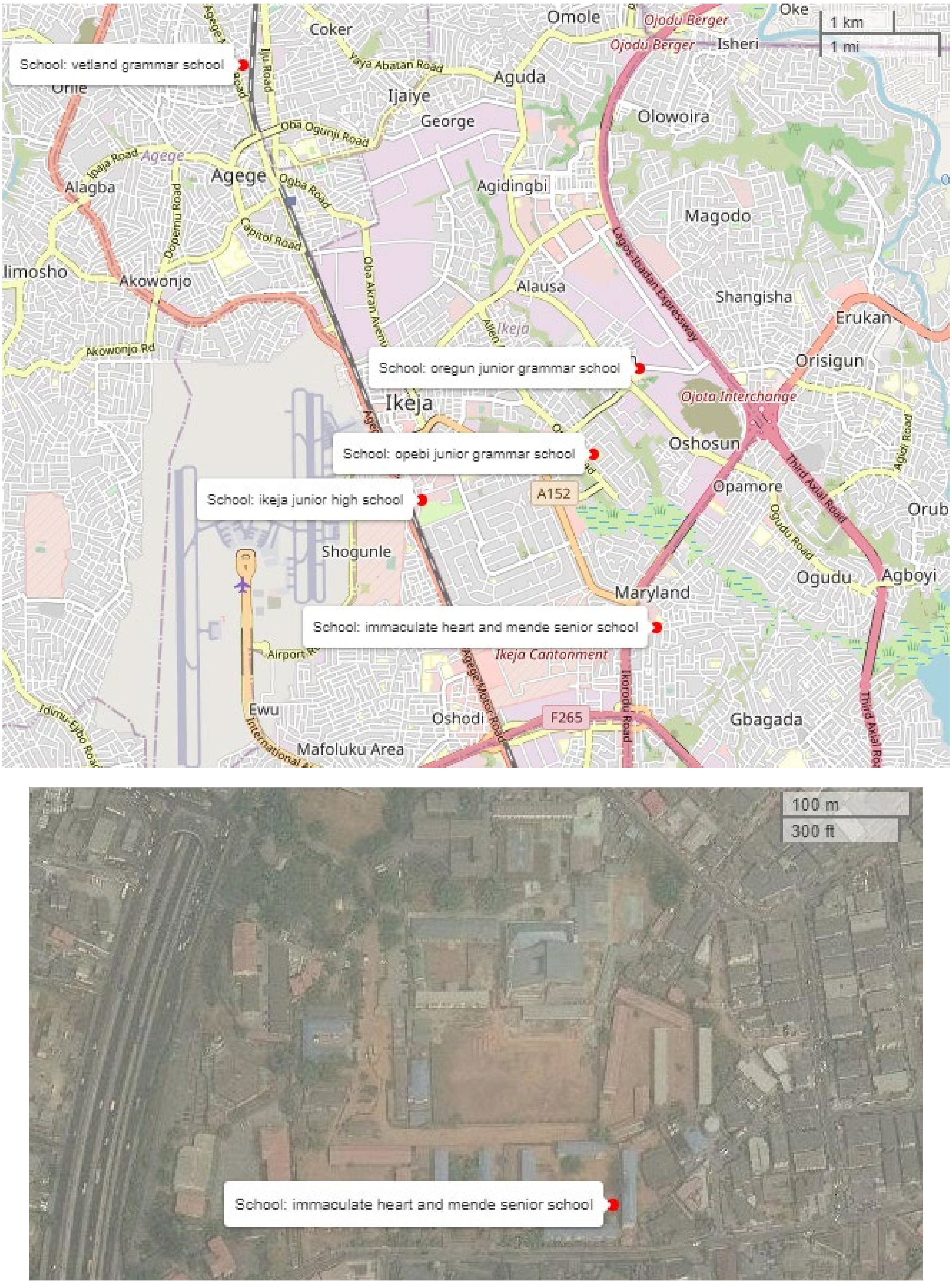

Figure S1.5: Location of six schools with participating students in Lagos, Nigeria and satellite image of an example of typical unpaved school grounds in Lagos.

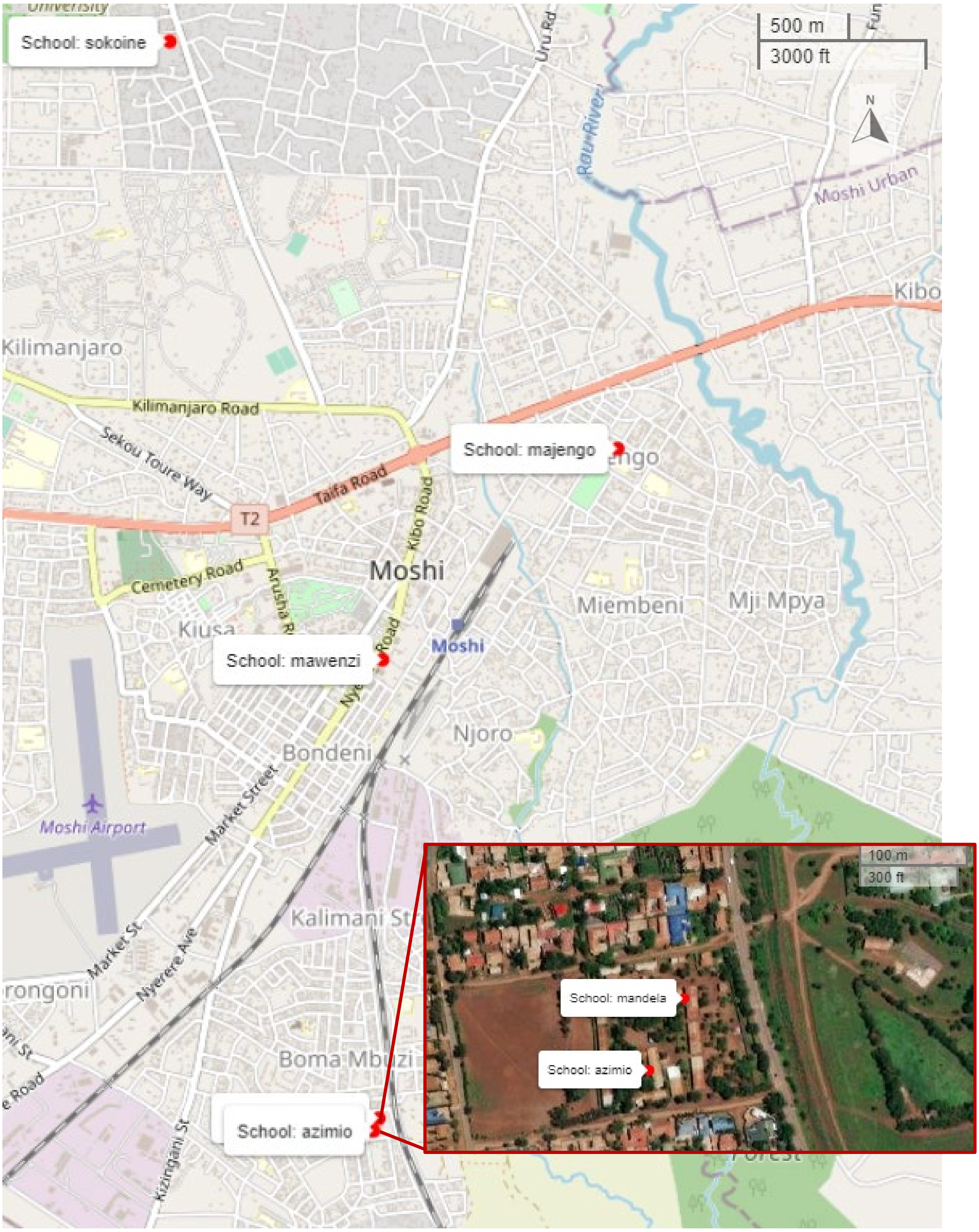

Figure S1.6: Location of five participating schools in Moshi, Tanzania and satellite image of typical school grounds in Moshi, showing Mandela and Azimio primary school.

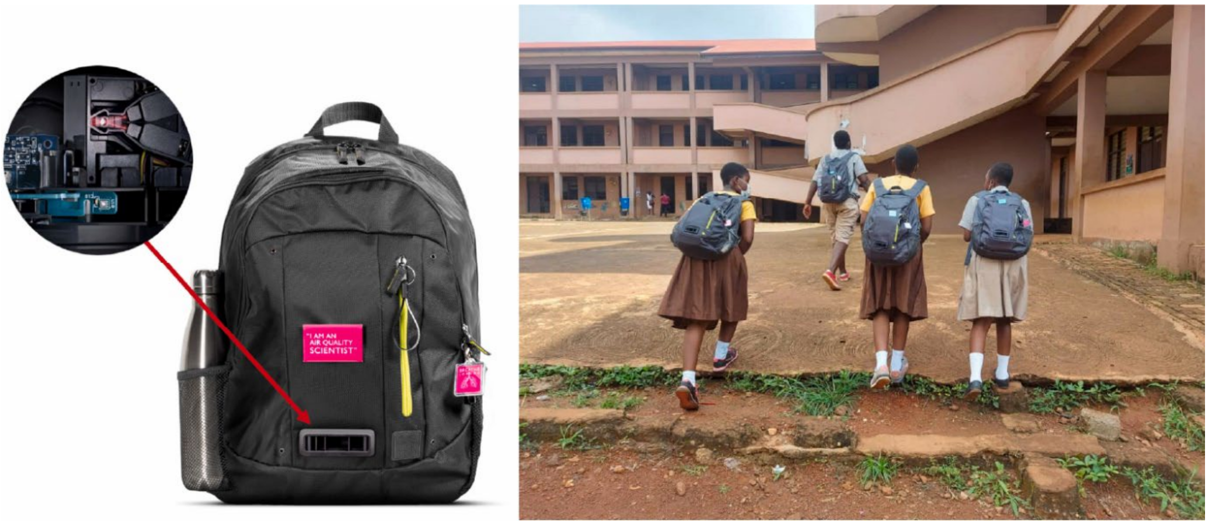

Figure S1.7: Photographs of the backpack used with an air pollution monitoring sensor and example of deployed backpacks with school children in Kumasi, Ghana.

2. Monitor co-location information

The monitor measured PM_{10}, $PM_{2.5}$ and NO_2. The PM sensor was a Sensirion SPS030 optical particle counter (OPC) (Sensirion AG, Switzerland). The NO_2 sensor was a Sensirion metal-oxide electrochemical sensor (Sensirion AG, Switzerland).

All sensors were calibrated and scaled by co-locating all sensors to a regulatory monitor prior to deployment. Typically, co-location should be conducted in the location of the field campaign to correct sensors to local pollution characteristics, however due to the scarcity of available regulatory monitors in sub-Saharan Africa, in this study it was completed in London, UK. Before the fieldwork, all of the backpacks were placed at the London Air Quality Network reference monitoring station at Honor Oak Park[1] and run continuously from 19 to 23 March 2021.

Deming regression was applied to co-location data from each backpack in comparison with matched reference data to produce a correlation factor (R^2), scaling factor and zero offset. For $PM_{2.5}$, all R^2 values were >0.90, indicating very good sensor agreement with the reference monitor. The mean (standard deviation) scaling factor and offset was 0.90 (0.04) and −1.60 (0.17) respectively, demonstrating good accuracy and precision. For PM_{10}, all R^2 values were >0.78, indicating good sensor agreement with the reference monitor. The mean (standard deviation) scaling factor and offset was 0.71 (0.04) and −2.50 (0.38). Calibration results for the NO_2 sensors showed relatively poor correlation against reference monitor results (R^2 from 0.00 to 0.41) so these exposure measurements could not be reliably scaled and therefore these results are not presented.

3. Ethical information

This study has been reviewed by the Queen Mary University of London Ethics of Research Committee in the UK. Each collaborating African centre received ethical approval for the study locally with:

Blantyre, Malawi: The College of Medicine Research Ethics Committee, University of Malawi, Reference No: P.04/21/3301.

Durban, South Africa: Biomedical Research Ethics Committee of University of KwaZulu Natal, Reference No: BREC/00002420/2021 and Department of Basic Education KwaZulu Natal and Department of Health KwaZulu Natal.

Harare, Zimbabwe: The City Health Ethics Board, Joint Research Ethics Committee, the Medical Research Council of Zimbabwe, Reference No: MRCZ/A/2415 and the Research Council of Zimbabwe.

Kumasi, Ghana: Committee on Human Research, Publication and Ethics a joint committee of the School of Medical Science, Kwame Nkrumah University of Science and Technology, Reference No: HRPE/AP /030/22.

Lagos, Nigeria: Lagos State Government and Lagos State University Teaching Hospital Health Research and Ethics Committee, Reference No: LREC/06/10/1484.

Moshi, Tanzania: National Health Research Ethics Review Committee, Reference No: NIMR/HQ/R.8c/Vol.I/928 and Kilimanjaro Christian Medical University College - College Research Ethics Review Committee, Reference No: 2489.

Kampala, Uganda: Mulago Hospital Research Ethics Committee, Reference No: MHREC2056, and Uganda National Council for Science and Technology number, Reference No: HS1695ES.

4. **Example of before monitoring questionnaire**

acacia achieving control of asthma in children in africa

CAPPA Questionnaire

Queen Mary University of London

Thank you very much for agreeing to fill in this questionnaire! A facilitator will be with you to answer any question you may have.

Section 1: Personal details

1. **Date of birth (DD/MM/YYYY):** *(digital: drop down menu)*

2. **Are you male or female?**
   - ○ Male
   - ○ Female

3. **Which school class or school year are you in?** ______________

4. **How would you describe your ethnicity?** *(digital: drop down menu with follow up please specify for 'mixed race' and 'other')*
   - ○ Black
   - ○ White
   - ○ East Asian, such as Chinese, Japanese, or Korean
   - ○ South Asian, such as Indian, or Bangladeshi
   - ○ Mixed race, please specify: ______________________
   - ○ Other, please specify: ___________________________

5. **Has a doctor, pharmacist, nurse, or healthcare worker ever said that you suffer from asthma?**
   - ○ Yes
   - ○ No
   - ○ Don't know

6. **Has a doctor, pharmacist, nurse, or healthcare worker ever said that you suffer from any of the following conditions?** (select all that apply)
   - ○ Atopic dermatitis or Eczema
   - ○ Hay fever and, or pollen allergy.

   Other allergies, please specify: _____________________________________
   - ○ Don't know

CAPPA questionnaire v1.0, 27/11/2020

The questionnaire was based on work from the School-based Asthma Project, funded by the NIHR (National Institute for Health Research) CLAHRC (Collaboration for Leadership in Applied Health Research and Care) North Thames. The license for the Asthma Control Test (ACT) was donated to the ACACIA/CAPPA study by the NIHR CLAHRC North Thames as part of the initial work.

7. **Have you ever been treated for Tuberculosis/TB?**
   - ◯ Yes
   - ◯ No
   - ◯ I'd rather not say
   - ◯ Don't know

8. **Have you ever had COVID-19?**
   - ◯ Yes
   - ◯ No
   - ◯ Prefer not to say
   - ◯ I don't know

If yes,

9. **Was COVID-19 confirmed by a test?**
   - ◯ Yes
   - ◯ No
   - ◯ I don't know

If yes,

10. **Have you ever been admitted to hospital with COVID-19?**
    - ◯ Yes
    - ◯ No
    - ◯ I don't know

Section 2: Asthma Control Test and GINA

(if ticked that asthma has been diagnosed)

1. **Do you have a reliever or rescue inhaler (usually blue or yellow, and using Salbutamol)?**

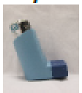
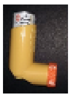

- Yes
- No

2. **In the past 4 weeks, how much of the time did your asthma keep you from getting as much done at work, school or home?**
- All of the time
- Most of the time
- Some of the time
- A little of the time
- None of the time

3. **In the past 4 weeks, have you had asthma symptoms during the day more than twice a week?**
- Yes
- No

4. **In the past 4 weeks, how often have you had shortness of breath?**
- More than once a day
- Once a day
- 3 to 6 times a week
- Once or twice a week
- Not at all

5. **In the past 4 weeks, how often did your asthma symptoms (wheezing, coughing, chest tightness, shortness of breath) wake you up at night or earlier than usual in the morning?**
- 4 or more nights a week
- 2 to 3 nights a week
- Once a week
- Once or twice (in 4 weeks)
- Not at all

If you have a reliever or rescue inhaler

6. **In the past 4 weeks, how often have you used your reliever inhaler (usually blue)?**
- 3 or more times per day
- Once or twice per day
- 3 times per week
- 2 times per week
- Once a week or less
- Not at all

7. **How would you rate your asthma control during the last 4 weeks?**
- Not controlled at all
- Poorly controlled
- Somewhat controlled
- Well controlled
- Completely controlled

Section 3: Medicines

1. **Do you take any medications for your asthma or wheeze?**

○ Yes

○ No

If yes,

2. **What type of medications do you usually use for your wheezing or asthma?**

Reliever Medicines						
Blue inhaler<br>- Subumol,<br>- Salbutamol,<br>- Ventolin<br>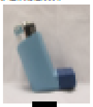<br>☐	Yellow inhaler<br>- Albuterol,<br>- Proventil<br>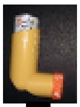<br>☐	Green inhaler<br>- Salmeterol<br>- Serevent<br>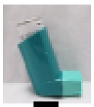<br>☐	Bricanyl<br>- Terbutaline DPI<br>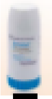<br>☐	Combivent HFA<br>- Salbutamol<br>- Ipratropium<br>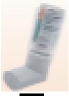<br>☐	Duovent<br>- Feneterol<br>- Ipratropium<br>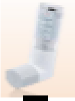<br>☐	Levosalbutamol Syrup<br>- Levolin<br>☐

Reliever Medicines			Preventer Medicines			
Ventolin tablets<br>- Salbutamol<br>☐	Ascoril Syrup<br>☐	Salbutamol syrup<br>☐	Brown inhaler<br>- Becasone,<br>- Beclometasone<br>- Becotide<br>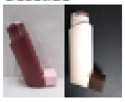<br>☐	Red inhaler<br>- Ciclesonide<br>- Alvesco<br>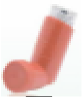<br>☐	Purple inhaler<br>- Fluticasone/ Salmeterol<br>- Seretide<br>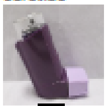<br>☐	Red/white inhaler<br>- Budesonide/ Formoterol<br>- Symbicort<br>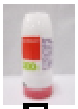<br>☐

Preventer Medicines						
Brown/white inhaler<br>- Budesonide<br>- Pulmicort<br>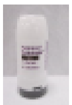<br>☐	Orange inhaler<br>- Fluticasone<br>- Flixotide<br>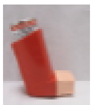<br>☐	Flixotide accuhaler DPI<br>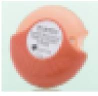<br>☐	Seretide Accuhaler<br>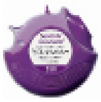<br>☐	Asthmanex twisthaler<br>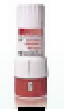<br>☐	Ciclovent, generic of Ciclesonide<br>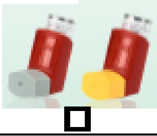<br>☐	Steroid tablets:<br>- Prednisolone<br>☐

Preventer Medicines				Other Medicines		
Theophylline tablets or Nuelin SA<br>☐	Ventolin tablets<br>- Salbutamol<br>☐	Franol, Asthmanol<br>- Ephedrine theophylline<br>☐	LTRA, Montelukast tablets<br>- Topraz,<br>- Singulair,<br>- Sintrine,<br>- Monte-Air<br>☐	Cough syrup<br>☐	Complementary, herbal, or alternative medicines<br>☐	Nebulizing<br>☐

Other Medicines
☐ I have other medicine, Please name: ______________________________

Section 8: Smoking, Home living, and Exposure

EXPOSURE

	Yes	No	Don't know
1. Is the main cooker in your home usually used outside?	Yes	No	Don't know
2. Is the main cooker in your home electric?	Yes	No	Don't know
3. Do you take part in cooking food at least once a week?	Yes	No	Don't know
4. Does the road nearest to your home have cars, buses, motor bikes or trucks driving along it all the time?	Yes	No	Don't know
5. Do you have any animals or pets living with you at home?	Yes	No	Don't know
6. Do you sometimes see or smell waste burning from a dump-site when at home?	Yes	No	Don't know
7. Do you sometimes breathe in smoke from bush fires?	Yes	No	Don't know
8. Does anyone living at your home use cleaning spray?	Yes	No	Don't know
9. Does anyone living at your home use insecticide spray?	Yes	No	Don't know
10. Does anyone living at your home use deodorant spray?	Yes	No	Don't know
11. Does anyone at your home burn mosquito coils?	Yes	No	Don't know
12. Does anyone at your home burn incense?	Yes	No	Don't know
13. Does anyone at your home burn crop residue, for example to heat a cooker.	Yes	No	Don't know
14. Is your home sometimes air conditioned?	Yes	No	Don't know
15. Can you sometimes see or smell a brick burning site from your home?	Yes	No	Don't know
16. Do you sometimes light your home with something else than electric light?	Yes	No	Don't know

If the main cooker in your home IS NOT electric

17. What type of cooker is the main stove at your home? Tick all that apply.

☐ Gas cooker, or mixed gas and electric	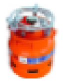 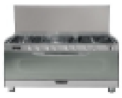
☐ Open fire or three stone stove	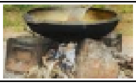
☐ Cooker using solid fuel, like coal or wood	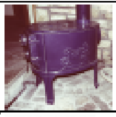 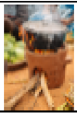
☐ Cooker using kerosene, oil, or another liquid	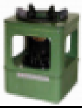 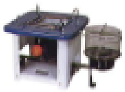
☐ Solar cooker	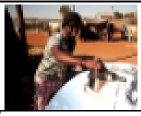
☐ Others: ______________________________	
☐ Don't know	

If you DO NOT always use electric lights,

18. What sort of non-electric lighting do you use? Tick all that apply.

☐ Candle (wax)	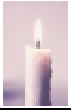
☐ Open fire	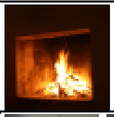
☐ Kerosene lamp	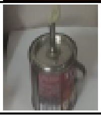 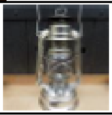
☐ Oil wick lamp	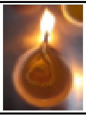

☐ Solar lantern	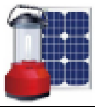
☐ Torch	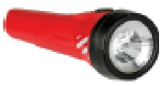
☐ Rechargeable lamp	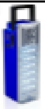 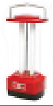 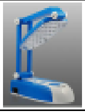
☐ Others: ______________________________	
☐ Don't know	

SMOKING

19. Have you ever smoked cigarettes or tobacco?

- Yes
- No
- I'd rather not say

If yes,

20. Have you smoked cigarettes or tobacco in the last month?

- Yes
- No

If yes to smoking in the last month:

21. How often do you smoke cigarettes or tobacco?

- Every day
- 5-6 days a week
- 3-4 days a week
- 1-2 days a week
- Less than once a week

22. Does anyone else smoke cigarettes or tobacco inside your home at least once a week?

- Yes
- No
- I'd rather not say

5. Example of participant daily diary template

CAPPA (Children's Air Pollution Profiles in Africa) - Study Diary

Version 1.1, 09/03/2021

For staff use only:
Participant ID:__________________, MONDAY Date (DD/MM/YYYY):______________________, Researcher:_______________

Evening Peak Flow: **Reading 1:** ______________ **Reading 2:** ______________ **Reading 3:** ______________

Did you have any of these symptoms/problems today? (please tick ✓)

	Yes:	No:
I used reliever inhaler	☐	☐
I had asthma symptoms such as shortness of breath, tight chest, coughing or wheezing	☐	☐
I woke at night with asthma symptoms	☐	☐
I feel like I can't keep up with my normal day-to-day activities	☐	☐
I have a cold or chest infection	☐	☐

How did you get *from* school today? (tick relevant boxes ✓)

Walking	☐	Car	☐	Minibus	☐	Train	☐
Bicycle	☐	Bus	☐	Motorbike or Scooter	☐	Tuk-tuk or cycle rickshaw	☐
Other:							

Were you near any of these situations today? (tick relevant boxes ✓)

**Waste burning dump site** 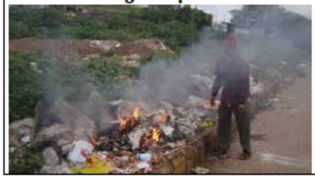	☐	**Construction site** 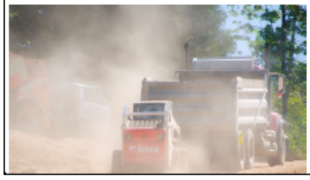	☐	**Someone smoking next to you** 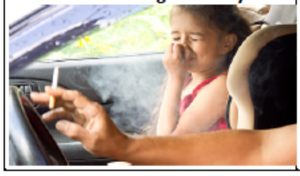	☐
**Brick burning site** 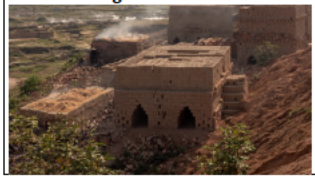	☐	**Bush fires** 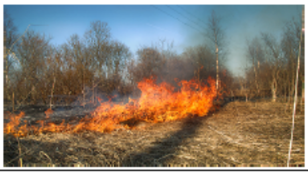	☐	**Smoke from electricity generator** 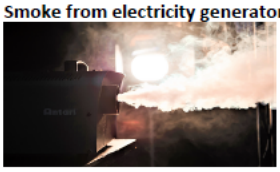	☐

**I wasn't near any of these situations:**	☐

Did you do any of this today? (tick relevant boxes ✓)

**Helping with cooking** 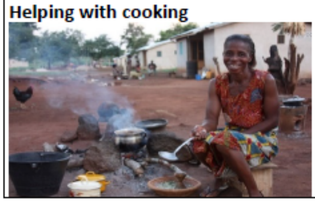	☐	**Direct contact with animals** 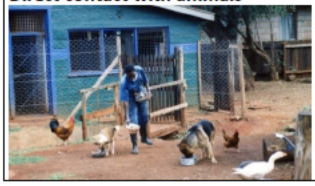	☐	**Smoking** 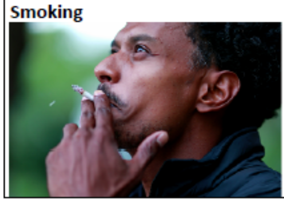	☐

**I didn't do any of this today:**	☐

6. Identifying participant microenvironments using GPS coordinates

The GPS coordinates were used to determine the type of microenvironment each participant was in. The primary objective of this analysis was to identify when the participant was at school, at home or commuting. The use of GPS coordinates to determine activities has been demonstrated in several papers previously[2–4], however the method has not been widely used in air pollution exposure studies.

The first part of GPS processing was to clean the GPS data, as satellite signals can get lost or drift, particularly in densely packed street canyons, in buildings or underground[5] . All GPS analyses were conducted in R statistics using libraries ‘geosphere’[6], ‘lubridate’[7] and ‘zoo’[8]. When the GPS signal was lost, points were interpolated between the last two known points if missing data was less than 45 minutes. If longer than 45 minutes GPS coordinates were repeated from the last known location. If GPS coordinates were lost for longer than 14 hours, GPS points were not interpolated (i.e. left as unknown). This long duration of time was justified as we did not want to remove GPS points where the participant was in the home and the GPS signal was lost, therefore the 14 hour rule was calculated from the typical time a child would be expected to be at home from 17:00 to 07:00 the next day. For participants with less than 24 hours of GPS information after interpolation, these points were all tagged as unclassified as comparisons between microenvironments could not be fairly compared.

The next step in the process was to run a spatial and temporal clustering algorithm on each participants data which is based on Spatio-Temporal Density-Based Spatial Clustering of Applications with Noise (ST-DBSCAN)[9], this identified clusters for points that were within 50m and 30 minutes of each other, with the minimum number of points required to make cluster being 30. This process followed a previous paper using ST-DBSCAN[10]. This resulted in each participant having between 2 to 25 clusters, which largely depended on the number of GPS coordinates that were available and how mobile the participant was during the monitoring week (Figure S6.1). Importantly this process also identified points which were not in a cluster, which provided a basis for identifying participants ‘move’ points.

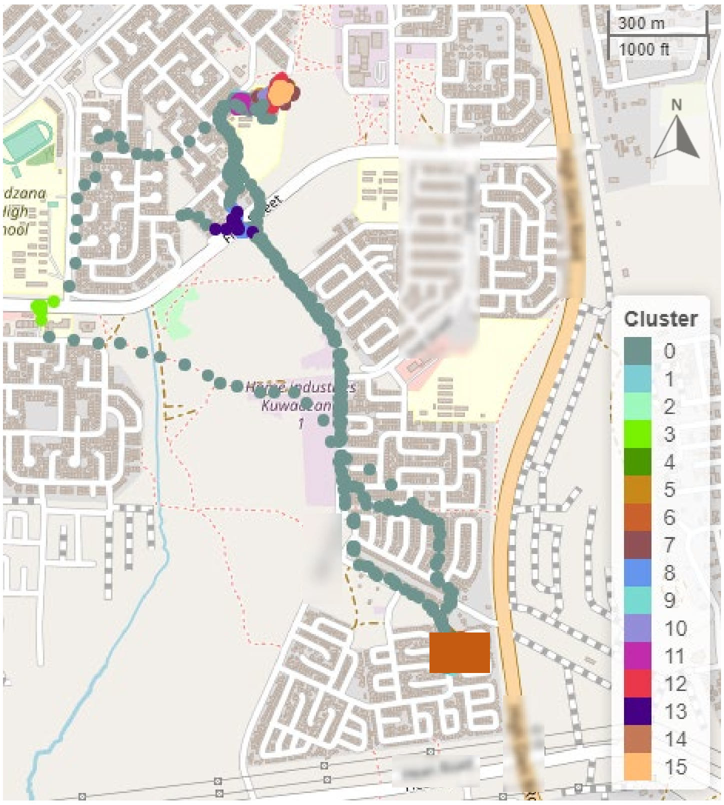

Figure S6.1: Example of a participant with ST-DBSCAN clustering algorithm run on GPS coordinates. In this example 15 clusters are formed. A cluster is formed for points that are within 50m and 30 minutes of each other. Any point that is not within a cluster is labelled as “0”.

After clusters were identified the next step was to identify when the participants were travelling. Speed was calculated between each one-minute point, additionally a rolling speed over 5-minutes was calculated. Using these consecutive points, stop and travel activities were identified, where 5 minutes of consecutive points of a rolling average 5-minute speed less than 1.6 km/hr was identified as a stop. This speed has previously been used to identify stop points previously[5]. All remaining points were labelled as ‘move’.

Consecutive move points were labelled as a trip. A first round of trips were identified using the rule described above but went through a number of filtering stages.

1) Any trip that was less than 5 minutes was tagged as a stop as we couldn’t be sure this was an actual trip and not GPS noise, this may have removed a few participants who lived next to school but this was a small proportion of the monitored population.
2) To address misclassification of commute trips which were due to GPS noise, any trip which had over 80% of points identified within a cluster were all labelled as stop as it was determined this was movement or drift of GPS coordinates at a fixed location and was not a trip (Figure S6.2).
3) The next step in filtering looked at any trip which had greater than 50% of points which were tagged as a cluster and identified the commute trip where there was greater than 5 consecutive minutes of points that were not identified as a cluster. The points in the clusters of these trips were labelled as stops (Figure S6.3).
4) Finally, if the end of a trip was within 15 minutes of the start of another trip, these were combined to make one larger trip.
5) The final step to identify travel was to visually inspect each participant’s trips on a map (Figure S6.4). Some participants trips were required to be manually removed as the rules-based algorithm did not correctly identify trips. The manual adjustment was typically due to GPS drift.

These filtering processes were conducted through trial and error and variations of these rules were run to produce optimal results.

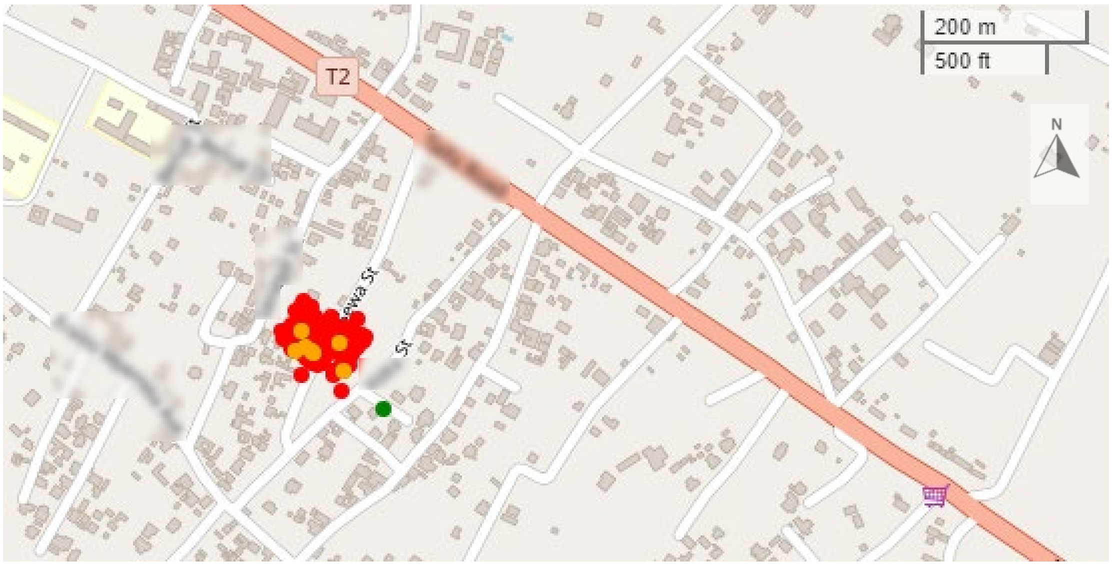

Figure S6.2: Example of a trip which was identified with > 80% of points within a cluster. The red points are stop points, orange points are cluster points identified as a trip while the green point is identified as a trip that is not included in a cluster.

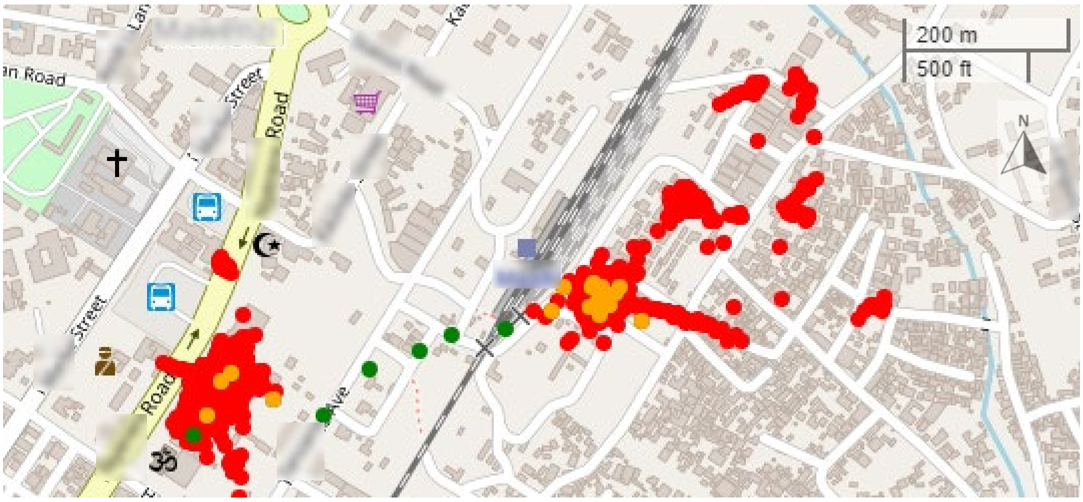

Figure S6.3: Example of a trip which was identified with > 50% of points within clusters. The red points are stop, orange points are cluster points identified as a trip while the green points are identified as a trip that is not included within a cluster. This misclassification occurs because the trip incorporates too much of the participants start and end points, so these trips are trimmed to only include where the participant is moving.

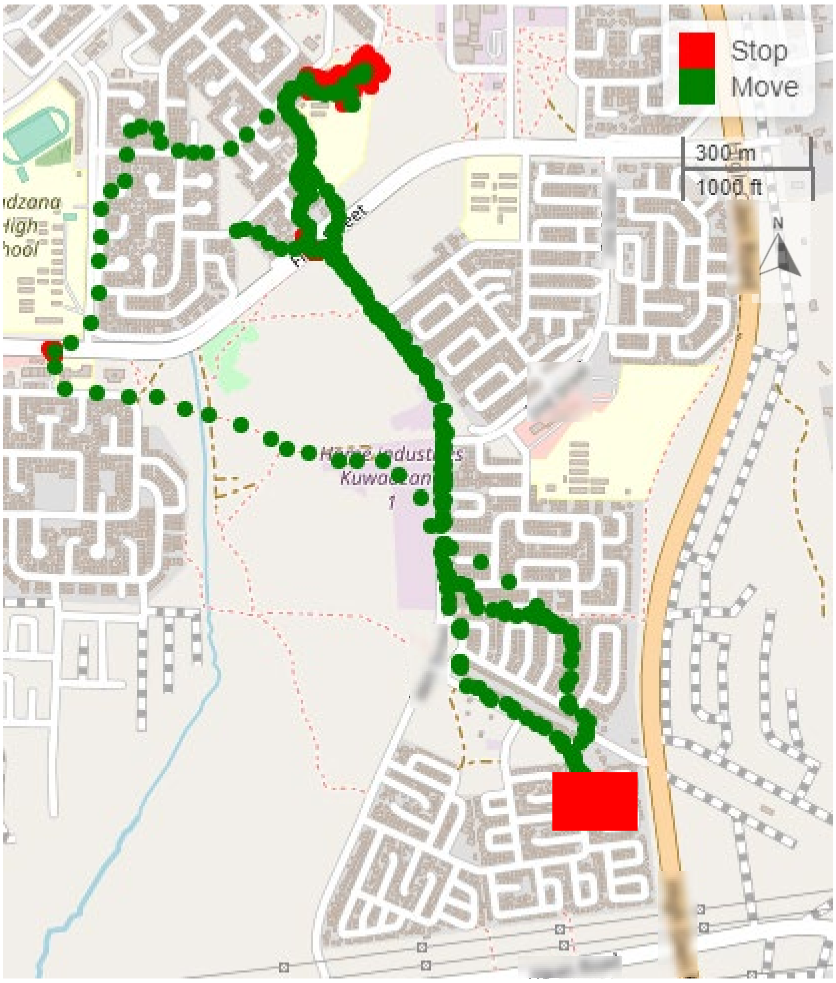

Figure S6.4: Example of stop and move map for a participant.

While this process identified travel points well, the next step was to identify the fixed locations of the clusters. The ST-DBSCAN process generally worked well in identifying these fixed points but resulted in a large number of individual clusters in the same location, due to a new cluster being formed once certain time and distance had been travelled. Instead, the DBSCAN algorithm was run to identify fixed locations, it is similar to ST-DBSCAN except it only clusters spatially and does not have a temporal element, therefore only one cluster is identified for each location.

To identify the participants fixed locations, first any point identified as a commute trip was removed from analysis. DBSCAN was run with an epsilon (eps) at 0.15, which represents euclidean distance and minimum number of points at 60 to form clusters for the whole monitoring period. An advantage of this approach compared to ST-DBSCAN is it better identifies locations that a participant frequently returns to during the monitoring period such as school and home environments as time is not a condition of clustering. The epsilon is calculated by first normalising the coordinate data, this makes it better for identifying fixed clusters as it accounts for the different distances between home and school locations for different participants.

Some of the participants home and school locations were so close together that only one cluster was formed. Therefore, for participants which resulted in a creation of a single cluster of greater than 75% of GPS points the DBSCAN algorithm was run again with a lower eps at 0.05 to try to separate those participants who lived close to their school. Approximately 2 to 3 clusters were identified per participant during the monitored school week (Figure S6.5), typically identifying school and home locations, however some participants had up to 8 clusters identified.

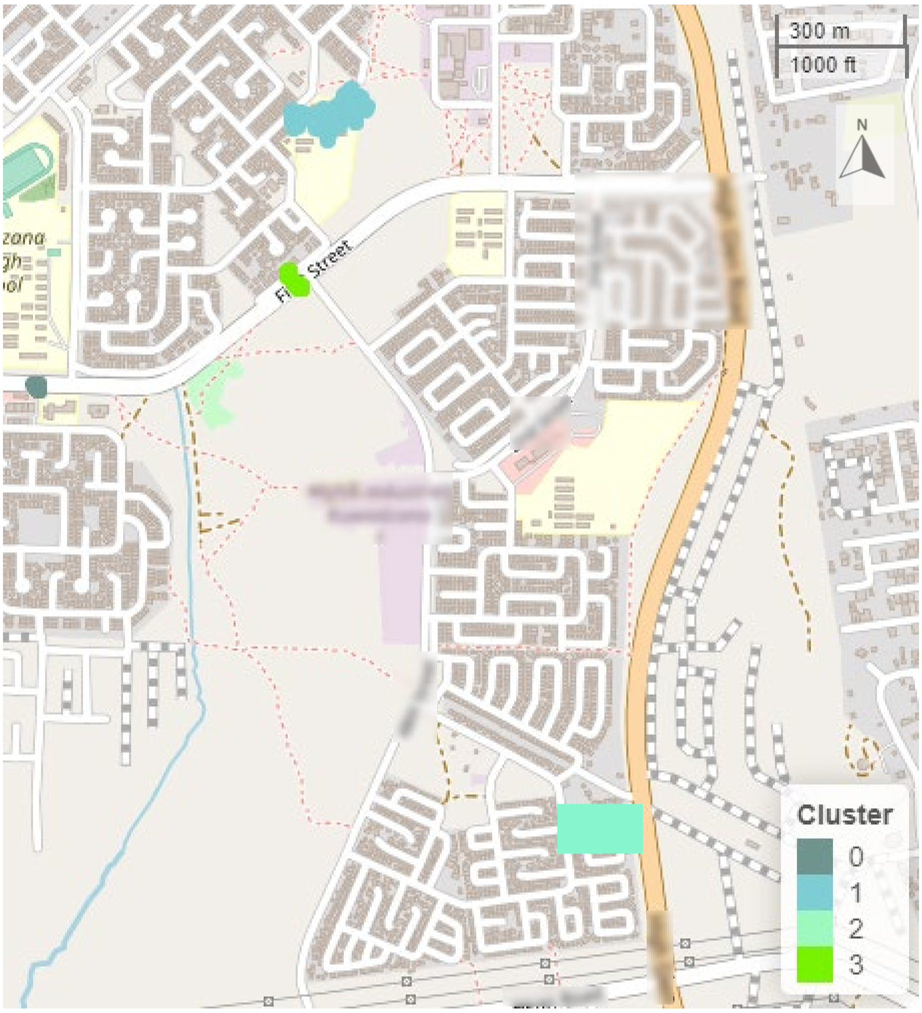

Figure S6.5: Example of a participant with DBSCAN clustering algorithm run on GPS coordinates without move points. In this example 3 clusters are formed. A cluster is formed for points that are within an epsilon (eps) at 0.15 and has a minimum number of points of 60. Any point that is not within a cluster is labelled as "0".

Once each participant had their individual clusters identified, each cluster was processed to produce an average coordinate. The average coordinate of each cluster was reverse geo-coded using the Nominatim API call to retrieve category (e.g. highway, amenity, building) and type (e.g. school, residential, market, hospital etc) information from the geocoding database OpenStreetMap.

From this information, the fixed locations were distilled into three types, school, residential and other. Specific locations such as hospital, place of worship, restaurant and other such locations were categorised as 'other' as during the school week these were visited too infrequently to make general exposure summaries and that the study was most interested in exposures at school and home where the children would spend most of their time. The residential location where the child spent most of their time was classified as 'home', while other residential points were classified as 'other residential'.

Microenvironments were therefore split into school, home, commute, other residential, other and unclassified. Points that were labelled as unclassified were where there weren't enough points to make a cluster or GPS coordinates were not recorded. Commute mode was determined by analysing commute points. The mode was first categorised by trip based on participant diary responses. These were then verified using mobility algorithms; any trip where average speed was > 5 km/hr was tagged as motorised, any trip less than 3 km/hr was tagged as walk. Trips between these speeds were tagged according to diary responses.

This output was then cleaned and checked by manually reviewing time activity histograms and maps (Figure S6.6 and S6.7). Participant's exposure information was removed during days where time was not spent at school, as there was uncertainty if the participant was with the monitoring backpack or they had left it at home on these days. Each child's GPS coordinates were checked for sensibility, such as a child travelling from home to school each day. Unless there was a reasonable explanation (i.e., if there was a public holiday or the school was closed due to COVID-19 restrictions), data were excluded for the day if the GPS coordinates showed that the participant did not take the bag to and from school.

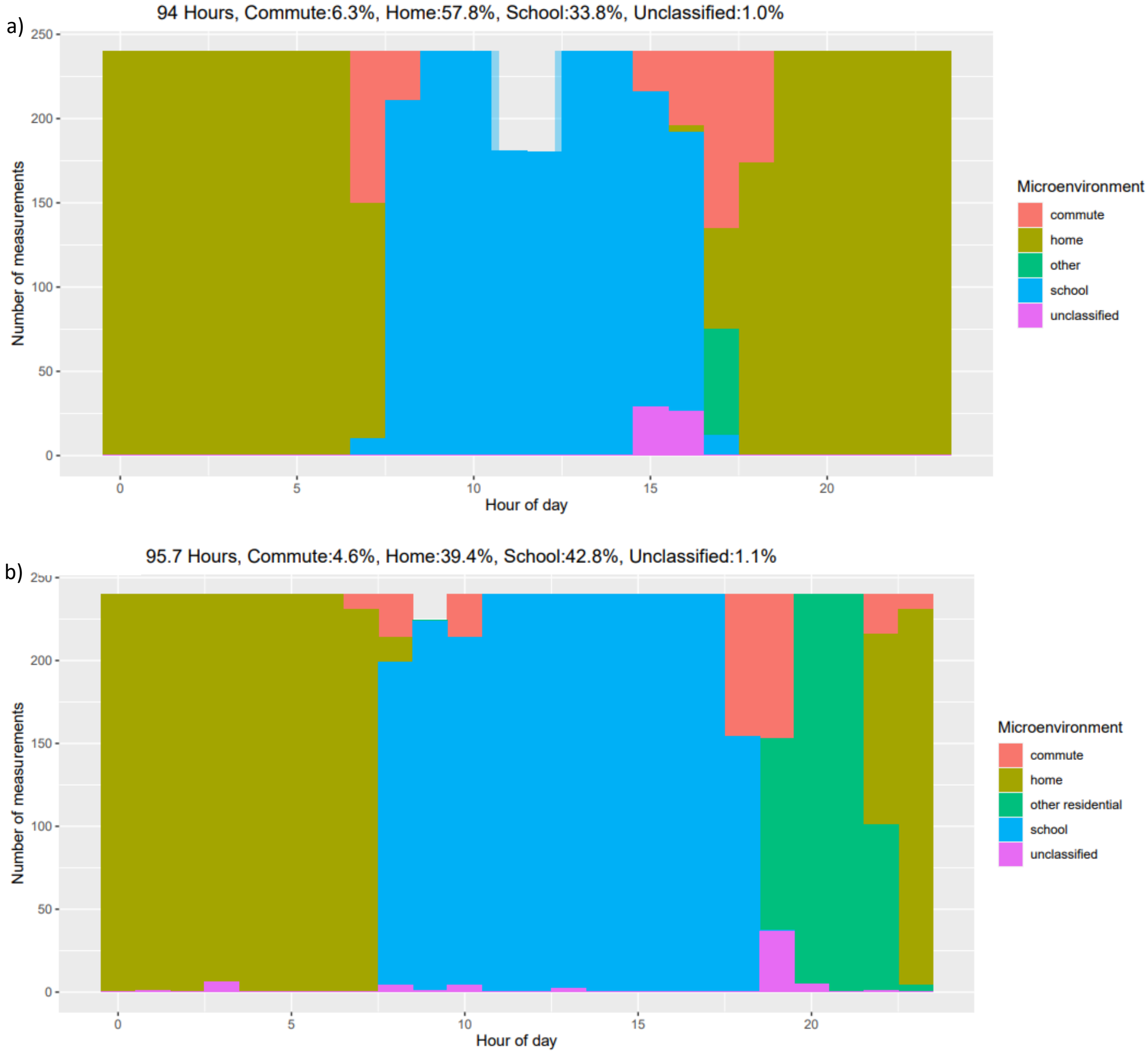

Figure S6.6: Examples of time activity histograms for participants in two different cities.

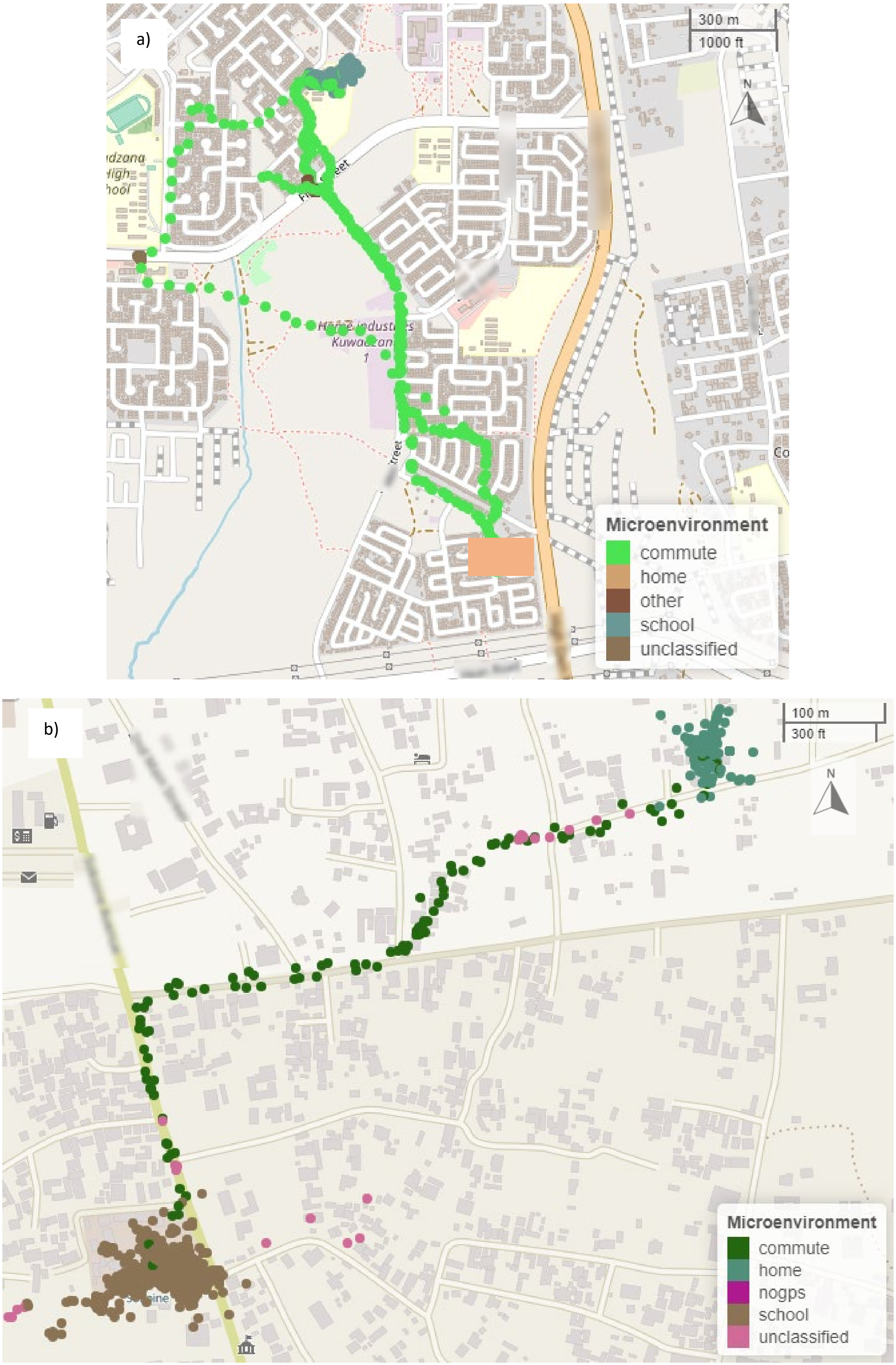

Figure S6.7: Examples of time activity map for participants in two different cities

7. Mixed effects models variables

Table S7.1: Variables tested in mixed effects models

Variable name	Included in final model	Information obtained from
Near waste burning site	Yes	Daily diary
Near construction site	Yes	Daily diary
Near electricity generator	No	Daily diary
Help with cooking	Yes	Daily diary
Contact with animals	Yes	Daily diary
Commute type	Yes	Daily diary
Someone smoking next to you	No	Daily diary
Near brick burning site	No	Daily diary
Use of backpack cover when raining	No	Daily diary
Near bush fires	No	Daily diary
Cooker location	Yes	Questionnaire
Cooker type	Yes	Questionnaire
Lighting use	Yes	Questionnaire
Smoking at home	Yes	Questionnaire
Sex	Yes	Questionnaire
Live near a busy road	Yes	Questionnaire
Use cleaning spray	No	Questionnaire
Use insect spray	No	Questionnaire
Use deodorant	No	Questionnaire
Burn coils	No	Questionnaire
Burn incense	No	Questionnaire
Burn crops	No	Questionnaire
Use air conditioning	No	Questionnaire
School grounds surface	Yes	Researcher observation
Day of week	Yes	Backpack
Mean daily air temperature (°C)	Yes	Worldmet[11]
Mean daily relative humidity (%)	Yes	Worldmet[11]
Mean daily wind speed (ms^{-1})	Yes	Worldmet[11]

8. Overall exposure summary

Of the 33 participants that were excluded from the analysis, 5 were removed from Blantyre due to incomplete questionnaires, 16 were from Durban (13 due to participants not taking backpacks to school as COVID disruptions were notable in this city and 3 due to equipment malfunction caused by external batteries disconnecting from the monitor), 7 from Harare due to equipment malfunction, 3 from Kumasi (2 withdrawing and 1 due to equipment malfunction), 1 from Lagos due to equipment malfunction and 1 from Moshi due to participant withdrawal.

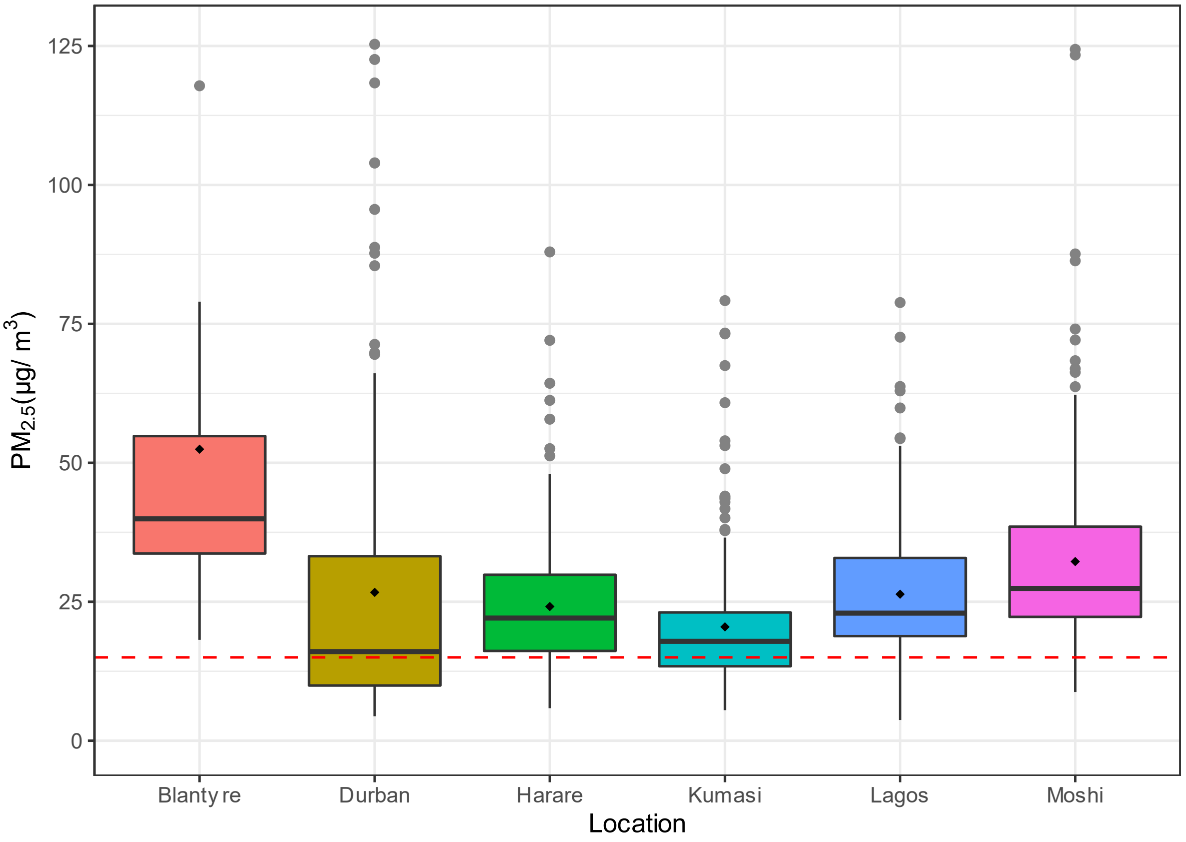

Figure S8.1: Asthmatic school children's $PM_{2.5}$ daily exposure across six locations in sub Saharan Africa. Bold horizontal black lines denote the median proportion; boxes extend from 25th to 75th percentile; vertical lines indicate 1.5 times the interquartile range from the 25th and 75th percentile; with dots being proportions outside the range of these values. Arithmetic means are represented by black diamonds. The dotted red line is the WHO 24-h guideline at 15 μg/m³. The y-axis excluded exposures >125 μg/m³, as a result, seven daily exposures are not presented (five days from Blantyre, one from Durban and one from Harare).

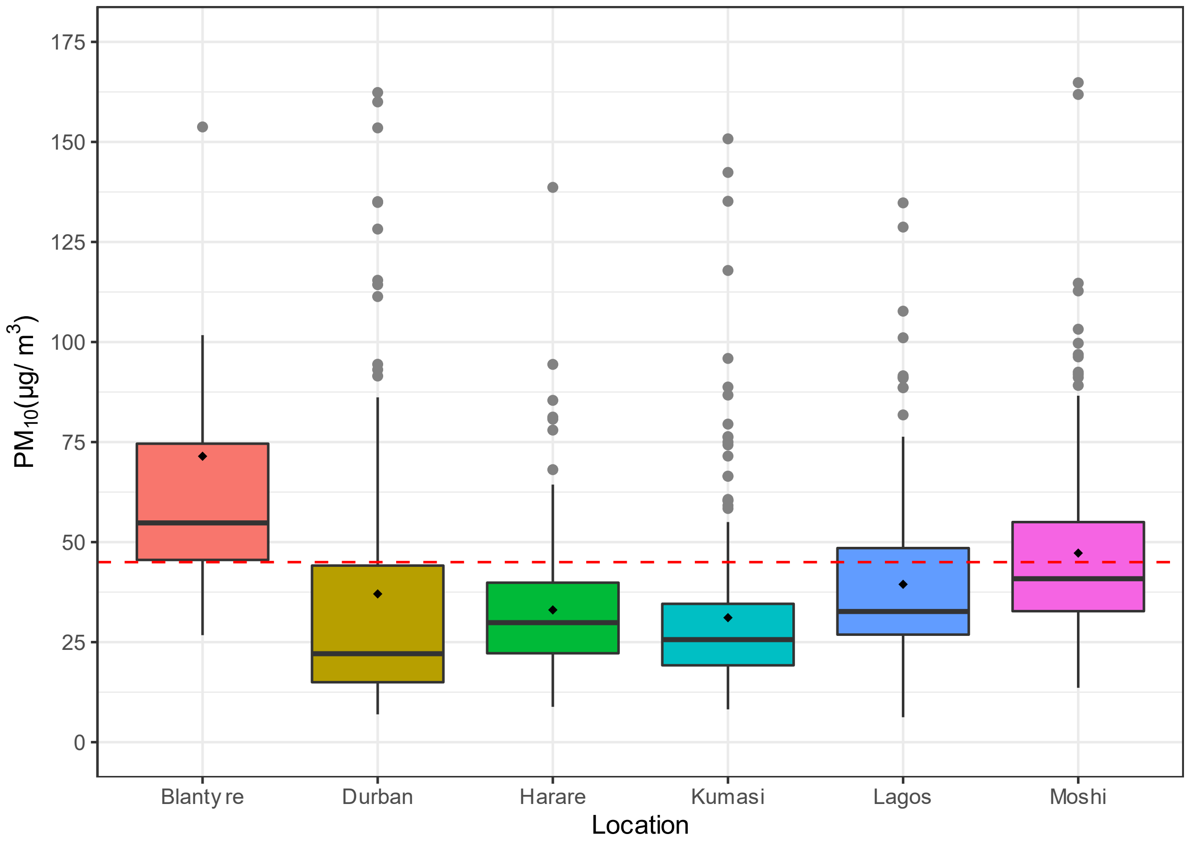

Figure S8.2: Asthmatic school children's PM_{10} daily exposure across six locations in sub Saharan Africa. Bold horizontal black lines denote the median proportion; boxes extend from 25th to 75th percentile; vertical lines indicate 1.5 times the interquartile range from the 25th and 75th percentile; with dots being proportions outside the range of these values. Arithmetic means are represented by black diamonds. The dotted red line is the WHO 24-h guideline at 45 µg/m^3. The y-axis excluded exposures >175 µg/m^3, as a result, seven daily exposures are not presented (five days from Blantyre, one from Durban and one from Harare).

There were small variations between $PM_{2.5}$:PM_{10} dail y ratios between locations with the median ratio ranging from 0.68 to 0.74 (Figure S8.3). Blantyre (0.74) and Harare (0.74) had significantly higher daily ratios compared to other locations ($p<0.01$) but were not significantly different to each other ($p = 0.30$). Durban had the next highest ratio (0.71) ($p<0.01$), while the other three cities (0.68 -0.70) had similar median daily ratios ($p>0.73$).

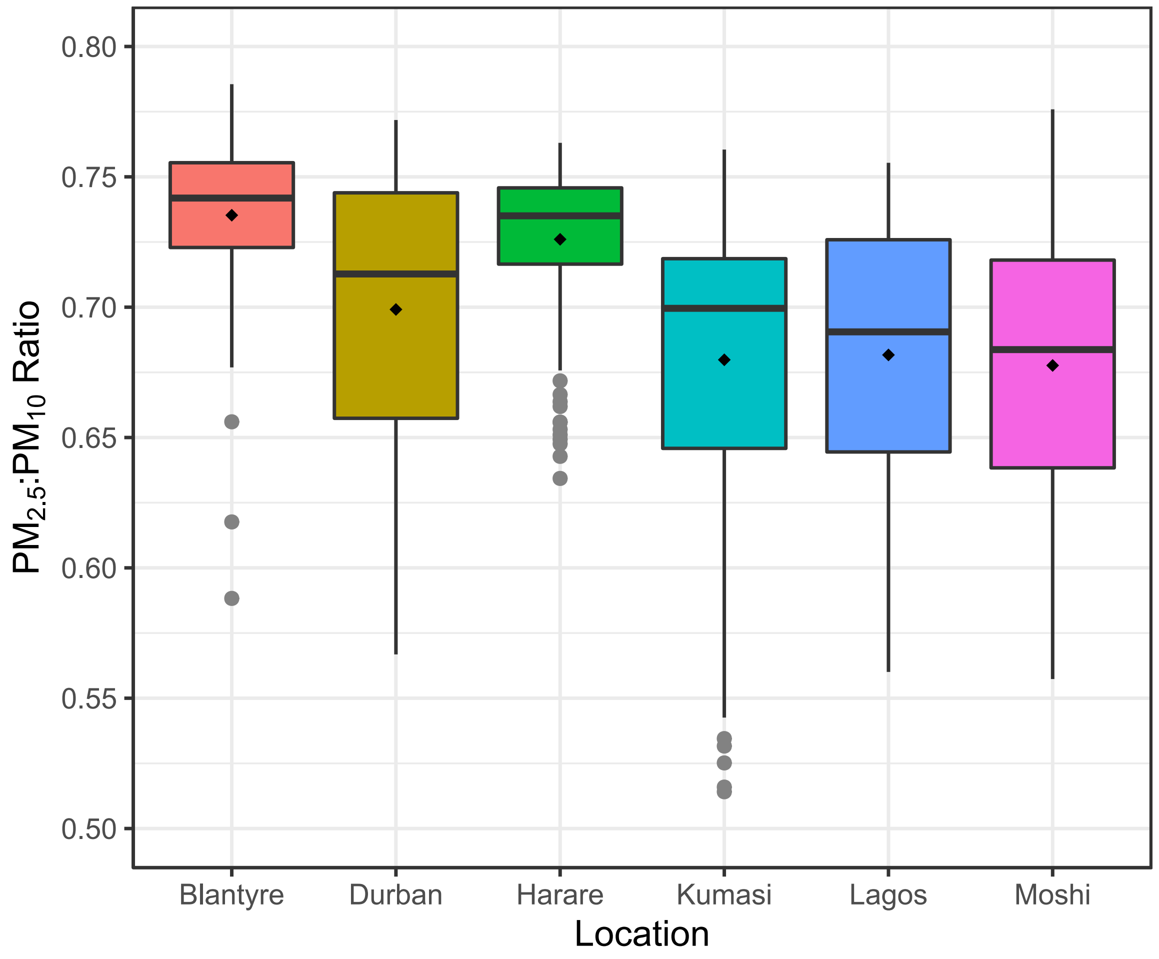

Figure S8.3: Asthmatic school children's $PM_{2.5}$:PM_{10} daily ratio across six locations in sub Saharan Africa. Bold horizontal black lines denote the median proportion; boxes extend from 25th to 75th percentile; vertical lines indicate 1.5 times the interquartile range from the 25th and 75th percentile; with dots being proportions outside the range of these values. Arithmetic means are represented by black diamonds.

Table S8.1: Asthmatic school children's personal PM_{10} daily exposure across six locations in sub Saharan Africa.

			PM_{10} exposure (µg/m^3)				
**Location**	**Number of participants**	**Days monitored**	**Minimum day**	**Median day**	**Maximum day**	**Arithmetic mean (SD) day**	**Geometric mean (SD) day**
Blantyre	24	81	26.7	55.9	363.6	71.5 (50.9)	62.2 (1.6)
Durban	47	180	7.0	22.1	344.3	37.1 (38.5)	27.0 (2.1)
Harare	43	162	8.8	29.9	138.7	33.1 (17.6)	29.4 (1.6)
Kumasi	61	237	8.2	25.6	150.8	31.1 (20.6)	27.0 (1.7)
Lagos	61	221	6.2	32.7	134.8	39.5 (19.6)	35.5 (1.6)
Moshi	61	228	13.6	40.9	176.8	47.3 (23.3)	43.0 (1.5)
**All**	**297**	**1109**	**6.2**	**32.8**	**363.6**	**40.3 (29.3)**	**33.8 (1.8)**

Table S8.2: Asthmatic school children's personal $PM_{2.5}$:PM_{10} daily ratio across six locations in sub Saharan Africa.

Location	Number of participants	Days monitored	$PM_{2.5}$:PM_{10} ratio Minimum day	Median day	Maximum day	Arithmetic mean (SD) day	Geometric mean (SD) day
Blantyre	24	81	0.59	0.74	0.79	0.74 (0.03)	0.73 (1.05)
Durban	47	180	0.57	0.71	0.77	0.70 (0.05)	0.70 (1.08)
Harare	43	162	0.63	0.74	0.76	0.73 (0.03)	0.73 (1.04)
Kumasi	61	237	0.51	0.70	0.76	0.68 (0.06)	0.68 (1.09)
Lagos	61	221	0.56	0.69	0.76	0.68 (0.05)	0.68 (1.08)
Moshi	61	228	0.56	0.68	0.78	0.68 (0.05)	0.68 (1.08)
**All**	**297**	**1109**	**0.51**	**0.71**	**0.79**	**0.69 (0.05)**	**0.69 (1.08)**

9. Microenvironment exposure results

Table S9.1: Asthmatic school children's personal $PM_{2.5}$ exposure measured in different microenvironments during four days monitoring across six locations in sub Saharan Africa. Participant numbers do not directly align with exposure data due to some monitors not recording a GPS position during monitoring.

		Median (minimum – maximum) $PM_{2.5}$ exposure (µg/m^3) in different microenvironments				
Location	Number of participants	Commute	Home	School	Other[a]	Unclassified[b]
Blantyre	24	43.2 (21.3 - 61.3)	48.0 (22.8 - 126.2)	32.3 (23.2 – 50.0)	28.6 (16.6 - 57.3)	159.7 (48.4 - 271.1)
Durban	47	16.3 (5.3 - 90.1)	20.9 (7.7 - 140.5)	11.9 (7.0 - 37.9)	15.5 (6.2 - 103.4)	19.5 (4.5 - 20.3)
Harare	41	25.4 (7.8 - 148.5)	22.7 (8.4 - 73.4)	16.4 (9.5 - 43.6)	11.3 (9.2 - 26.7)	25.0 (12.1 - 28.6)
Kumasi	61	28.3 (6.1 - 73.7)	14.2 (7.3 - 51.8)	19.6 (6.5 - 122.1)	17.9 (4.4 - 41.1)	16.9 (13.8 - 17.4)
Lagos	57	31.5 (13.5 - 86.5)	18.0 (5.6 - 52.9)	32.0 (10.9 - 104.4)	18.8 (4.4 – 51.0)	16.8 (12.3 - 37.4)
Moshi	59	31.6 (14.1 - 165.7)	23.6 (8.9 - 135.8)	33.1 (16.6 - 76.3)	28.9 (10.6 - 105.1)	26.1 (12.8 - 145.9)

[a]Only 5 participants had exposure in 'Other' microenvironment for Blantyre, 7 for Durban, 5 for Harare, 10 for Kumasi, 22 for Lagos and 17 for Moshi.
[b]Only 2 participants had exposure in 'Unclassified' microenvironment for Blantyre, 3 for Durban, 5 for Harare, 3 for Kumasi, 5 for Lagos and 14 for Moshi.

Table S9.2: Asthmatic school children's total time spent in different microenvironments during four days monitoring across six locations in sub Saharan Africa.

		Mean (SD) for hours monitored					
Location	Number of participants	Total	Commute	Home	School	Other[a]	Unclassified[b]
Blantyre	24	88.1 (10.4)	3.4 (2.3)	59.4 (13.4)	18.2 (5.7)	4.1 (5.3)	18.7 (1.5)
Durban	47	84.4 (15.0)	3.1 (2.1)	67.7 (13.4)	15.7 (7.2)	3.4 (2.2)	27.2 (9.4)
Harare	41	80.9 (22.0)	2.8 (2.2)	51.5 (16.2)	26.4 (8.4)	8.7 (5.7)	73.8 (21.9)
Kumasi	61	89.2 (12.3)	5.1 (2.6)	52.6 (9.1)	27.9 (5.5)	3.6 (3.7)	31.1 (15.2)
Lagos	57	86.1 (15.9)	4.5 (2.8)	50.7 (16.2)	20.6 (7.5)	8.2 (7.9)	85.7 (7.1)
Moshi	59	86.9 (18.1)	3.6 (2.2)	43.9 (14.0)	28.8 (9.1)	4.8 (4.4)	45.7 (24.4)

[a]Only 5 participants spent time in 'Other' microenvironment for Blantyre, 7 for Durban, 5 for Harare, 10 for Kumasi, 22 for Lagos and 17 for Moshi.
[b]Only 2 participants spent time in 'Unclassified' microenvironment for Blantyre, 3 for Durban, 5 for Harare, 3 for Kumasi, 5 for Lagos and 14 for Moshi.

Table S9.3: Asthmatic school children's personal $PM_{2.5}$:PM_{10} ratio measured in different microenvironments during four days monitoring across six locations in sub Saharan Africa.

Location	Number of participants	Median (minimum – maximum) $PM_{2.5}$:PM_{10} ratio in different microenvironments		
		Commute	Home	School
Blantyre	24	0.72 (0.66 - 0.77)	0.74 (0.67 - 0.77)	0.73 (0.69 - 0.76)
Durban	47	0.68 (0.58 - 0.74)	0.68 (0.61 - 0.75)	0.67 (0.56 - 0.75)
Harare	41	0.72 (0.65 - 0.75)	0.72 (0.61 - 0.75)	0.72 (0.63 - 0.75)
Kumasi	61	0.72 (0.64 - 0.75)	0.70 (0.62 - 0.74)	0.69 (0.58 - 0.73)
Lagos	57	0.73 (0.68 - 0.75)	0.72 (0.57 - 0.76)	0.69 (0.61 - 0.75)
Moshi	59	0.69 (0.58 - 0.75)	0.70 (0.60 - 0.75)	0.67 (0.58 - 0.73)

Table S9.4: Asthmatic school children's proportion of personal $PM_{2.5}$ total exposure and time spent in different microenvironments during four days monitoring across six locations in sub Saharan Africa.

Location	Number of participants	Proportion	Median (minimum – maximum) proportion (%) of total $PM_{2.5}$ exposure and time spent			
			Commute	Home	School	Other
Blantyre	22	$PM_{2.5}$ exposure	3.5% (0.2% - 13.8%)	76.0% (46.2% - 93.3%)	17.6% (4.1% - 31.0%)	1.3% (0.0% - 22.3%)
		Time spent	4.2% (0.3% - 10.1%)	72.1% (53.9% - 85.3%)	21.4% (8.7% - 34.0%)	2.0% (0.0% - 15.6%)
Durban	44	$PM_{2.5}$ exposure	2.2% (0.1% - 26.0%)	81.5% (61.7% - 98.2%)	11.6% (1.5% - 35.0%)	0.4% (0.0% - 15.6%)
		Time spent	3.2% (0.2% - 12.8%)	80.8% (62.6% - 92.9%)	14.8% (4.8% - 33.0%)	0.8% (0.0% - 6.9%)
Harare	38	$PM_{2.5}$ exposure	2.1% (0.4% - 22.2%)	68.9% (46.5% - 83.3%)	27.3% (11.7% - 44.4%)	0.9% (0.0% - 15.1%)
		Time spent	2.4% (0.5% - 11.0%)	64.7% (41.9% - 79.7%)	32.7% (17.6% - 42.6%)	1.1% (0.0% - 18.5%)
Kumasi	58	$PM_{2.5}$ exposure	8.0% (1.1% - 33.7%)	54.4% (10.6% - 84.5%)	35.9% (12.9% - 83.8%)	0.8% (0.0% - 32.1%)
		Time spent	6.2% (1.0% - 13.0%)	61.2% (44.0% - 72.7%)	31.8% (24.9% - 43.4%)	0.8% (0.0% - 14.9%)
Lagos	56	$PM_{2.5}$ exposure	6.8% (1.2% - 22.9%)	51.2% (20.0% - 77.5%)	38.6% (9.7% - 69.6%)	2.1% (0.0% - 35.5%)
		Time spent	5.3% (1.3% - 15.3%)	66.7% (30.0% - 76.5%)	25.8% (9.6% - 50.0%)	2.9% (0.0% - 43.6%)
Moshi	47	$PM_{2.5}$ exposure	4.9% (1.1% - 37.9%)	47.5% (12.3% - 86.8%)	45.7% (6.4% - 72.2%)	1.3% (0.0% - 38.7%)
		Time spent	3.9% (1.2% - 12.3%)	55.9% (35.6% - 71.9%)	37.1% (23.6% - 46.3%)	1.4% (0.0% - 20.1%)

10. Descriptive statistics on potential determinants of personal exposure

Table S10.1: Distribution of potential determinants of participant exposure on monitoring days by location

	Total days	Blantyre	Durban	Harare	Kumasi	Lagos	Moshi
Monitoring days	1109	81 (7%)	180 (16%)	162 (15%)	237 (21%)	221 (20%)	228 (21%)
Near waste burning site							
Yes	210 (19%)	26 (32%)	17 (9%)	48 (30%)	37 (16%)	22 (10%)	60 (26%)
No	899 (81%)	55 (68%)	163 (91%)	114 (70%)	200 (84%)	199 (90%)	168 (74%)
Near construction site							
Yes	94 (8%)	11 (14%)	24 (13%)	2 (1%)	5 (2%)	18 (8%)	34 (15%)
No	1015 (92%)	70 (86%)	156 (87%)	160 (99%)	232 (98%)	203 (92%)	194 (85%)
Near electricity generator							
Yes	55 (5%)	2 (2%)	1 (1%)	0	0	40 (18%)	12 (5%)
No	1054 (95%)	79 (98%)	179 (99%)	162 (100%)	237 (100%)	181 (82%)	216 (95%)
Help with cooking							
Yes	292 (26%)	39 (48%)	9 (5%)	9 (6%)	28 (12%)	73 (33%)	134 (59%)
No	817 (74%)	42 (52%)	171 (95%)	153 (94%)	209 (88%)	148 (67%)	94 (41%)
Contact with animals							
Yes	164 (15%)	32 (40%)	20 (11%)	6 (4%)	19 (8%)	21 (10%)	66 (29%)
No	945 (85%)	49 (60%)	160 (89%)	156 (96%)	218 (92%)	200 (90%)	162 (71%)
Commute mode							
Motorised	358 (32%)	0	57 (32%)	15 (9%)	145 (61%)	104 (47%)	37 (16%)
Walk	507 (46%)	64 (79%)	73 (41%)	105 (65%)	64 (27%)	68 (31%)	133 (58%)
Mixed mode	96 (9%)	7 (9%)	6 (3%)	12 (7%)	22 (9%)	29 (13%)	20 (9%)
No commute	98 (9%)	9 (11%)	43 (24%)	17 (10%)	5 (2%)	6 (3%)	18 (8%)
Unclassified	50 (5%)	1 (1%)	1 (1%)	13 (8%)	1 (0%)	14 (6%)	20 (9%)
Someone smoking next to you							
Yes	157 (14%)	11 (14%)	22 (12%)	4 (2%)	0	31 (14%)	89 (39%)
No	952 (86%)	70 (86%)	158 (88%)	158 (98%)	237 (100%)	190 (86%)	139 (61%)
Near brick burning site							
Yes	29 (3%)	8 (10%)	1 (1%)	0	1 (0%)	4 (2%)	15 (7%)
No	1080 (97%)	73 (90%)	179 (99%)	162 (100%)	236 (100%)	217 (98%)	213 (93%)
Use of backpack cover when raining							
Yes	114 (10%)	5 (6%)	4 (2%)	7 (4%)	28 (12%)	54 (24%)	16 (7%)
No	989 (90%)	76 (94%)	176 (98%)	155 (96%)	203 (88%)	167 (76%)	212 (93%)
Near bush fires							
Yes	32 (3%)	5 (6%)	7 (4%)	1 (1%)	6 (3%)	0	13 (6%)
No	1077 (97%)	76 (94%)	173 (96%)	161 (99%)	231 (97%)	221 (100%)	215 (94%)
Cooker location							
Inside	800 (72%)	38 (47%)	149 (83%)	140 (86%)	158 (67%)	171 (77%)	144 (63%)
Outside	286 (26%)	43 (53%)	15 (8%)	19 (12%)	79 (33%)	46 (21%)	84 (37%)
Not reported	23 (2%)	0	16 (9%)	3 (2%)	0	4 (2%)	0

	Total days	Blantyre	Durban	Harare	Kumasi	Lagos	Moshi
Cooker type							
Electric	442 (40%)	20 (25%)	136 (76%)	144 (89%)	60 (25%)	78 (35%)	4 (2%)
Gas	284 (26%)	0	32 (18%)	18 (11%)	83 (35%)	111 (50%)	40 (18%)
Gas and biomass	240 (22%)	0	8 (4%)	0	66 (28%)	22 (10%)	144 (63%)
Coal or wood (biomass)	71 (6%)	36 (44%)	0	0	24 (10%)	0	11 (5%)
Kerosene	23 (2%)	0	0	0	0	2 (1%)	21 (9%)
Open fire and biomass	41 (4%)	25 (31%)	0	0	4 (2%)	4 (2%)	8 (4%)
Not reported	8 (1%)	0	4 (2%)	0	0	4 (2%)	0
Alternative lighting use							
Electric only	365 (33%)	43 (53%)	24 (13%)	16 (10%)	175 (74%)	99 (45%)	8 (4%)
Candle	198 (18%)	19 (23%)	44 (24%)	85 (52%)	4 (2%)	15 (7%)	31 (14%)
Candle and other	183 (17%)	3 (4%)	47 (26%)	8 (5%)	15 (6%)	37 (17%)	73 (32%)
Kerosene lamp and other	92 (8%)	4 (5%)	8 (4%)	0	4 (2%)	10 (5%)	66 (29%)
Recharge, solar lamp or torch	239 (22%)	8 (10%)	33 (18%)	49 (30%)	39 (16%)	60 (27%)	50 (22%)
Not reported	32 (3%)	4 (5%)	24 (13%)	4 (2%)	0	0	0
Smoking at home							
No Smokers	880 (79%)	63 (78%)	111 (62%)	108 (67%)	222 (94%)	167 (76%)	209 (92%)
Presence of smokers	211 (19%)	18 (22%)	62 (34%)	54 (33%)	15 (6%)	43 (19%)	19 (8%)
Not reported	18 (2%)	0	7 (4%)	0	0	11 (5%)	0
Gender							
Female	663 (60%)	50 (62%)	123 (68%)	76 (47%)	155 (65%)	138 (62%)	121 (53%)
Male	446 (40%)	31 (38%)	57 (32%)	86 (53%)	82 (35%)	83 (38%)	107 (47%)
Live near a busy road							
Yes	808 (73%)	35 (43%)	120 (67%)	113 (70%)	171 (72%)	170 (77%)	199 (87%)
No	278 (25%)	46 (57%)	44 (24%)	49 (30%)	66 (28%)	44 (20%)	29 (13%)
Not reported	23 (2%)	0	16 (9%)	0	0	7 (3%)	0
Use cleaning spray							
Yes	437 (39%)	16 (20%)	122 (68%)	59 (36%)	116 (49%)	106 (48%)	18 (8%)
No	612 (55%)	65 (80%)	34 (19%)	103 (64%)	113 (48%)	87 (39%)	210 (92%)
Not reported	60 (5%)	0	24 (13%)	0	8 (3%)	28 (13%)	0
Use insect spray							
Yes	636 (57%)	39 (48%)	68 (38%)	73 (45%)	183 (77%)	136 (62%)	137 (60%)
No	407 (37%)	42 (52%)	80 (44%)	89 (55%)	46 (19%)	59 (27%)	91 (40%)
Not reported	66 (6%)	0	32 (18%)	0	8 (3%)	26 (12%)	0
Use deodorant							
Yes	718 (65%)	46 (57%)	109 (61%)	131 (81%)	185 (78%)	87 (39%)	160 (70%)
No	314 (28%)	35 (43%)	55 (31%)	28 (17%)	44 (19%)	84 (38%)	68 (30%)
Not reported	77 (7%)	0	16 (9%)	3 (2%)	8 (3%)	50 (23%)	0
Burn coils							
Yes	680 (61%)	39 (48%)	91 (51%)	137 (85%)	161 (68%)	107 (48%)	145 (64%)
No	405 (37%)	42 (52%)	85 (47%)	25 (15%)	72 (30%)	98 (44%)	83 (36%)
Not reported	24 (2%)	0	4 (2%)	0	4 (2%)	16 (7%)	0

	Total	Blantyre	Durban	Harare	Kumasi	Lagos	Moshi
Burn incense							
Yes	272 (25%)	12 (15%)	73 (41%)	24 (15%)	16 (7%)	38 (17%)	109 (48%)
No	753 (68%)	69 (85%)	72 (40%)	138 (85%)	213 (90%)	142 (64%)	119 (52%)
Not reported	84 (8%)	0	35 (19%)	0	8 (3%)	41 (19%)	0
Burn crops							
Yes	312 (28%)	22 (27%)	28 (16%)	82 (51%)	22 (9%)	63 (29%)	95 (42%)
No	715 (64%)	59 (73%)	116 (64%)	80 (49%)	203 (86%)	124 (56%)	133 (58%)
Not reported	82 (7%)	0	36 (20%)	0	12 (5%)	34 (15%)	0
Use air conditioning							
Yes	243 (22%)	16 (20%)	53 (29%)	16 (10%)	39 (16%)	97 (44%)	22 (10%)
No	850 (77%)	65 (80%)	115 (64%)	146 (90%)	194 (82%)	124 (56%)	206 (90%)
Not reported	16 (1%)	0	12 (7%)	0	4 (2%)	0	0
School grounds surface							
Loose dirt	648 (58%)	81 (100%)	0	0	163 (69%)	176 (80%)	228 (100%)
Packed dirt	65 (6%)	0	0	0	20 (8%)	45 (20%)	0
Broken paving	74 (7%)	0	74 (41%)	0	0	0	0
Paved	322 (29%)	0	106 (59%)	162 (100%)	54 (23%)	0	0
Day of week							
Monday	288 (26%)	21 (26%)	47 (26%)	42 (26%)	61 (26%)	61 (28%)	56 (25%)
Tuesday	279 (25%)	21 (26%)	45 (25%)	42 (26%)	60 (25%)	51 (23%)	60 (26%)
Wednesday	274 (25%)	19 (23%)	45 (25%)	39 (24%)	58 (24%)	56 (25%)	57 (25%)
Thursday	268 (24%)	20 (25%)	43 (24%)	39 (24%)	58 (24%)	53 (24%)	55 (24%)
Daily average air temperature (°C)	24.2 (4.2)	28.2 (4.2)	19.1 (1.9)	22.5 (4.8)	27.4 (2.0)	27 (1.2)	22.5 (2.2)
Daily average relative humidity (%)	67.7 (16.1)	44.4 (14.2)	72.1 (8.9)	54.8 (18.1)	77.1 (8.9)	82.2 (5.7)	58.9 (8.9)
Daily average wind speed (ms^{-1})	3.1 (1.3)	3.8 (1.3)	2.8 (0.8)	4.0 (1.2)	1.8 (0.6)	3.1 (0.8)	3.7 (1.5)

Data are n (%) or mean (SD)

Table S10.2: Summary statistics by participant for mean, minimum, median and maximum $PM_{2.5}$ daily exposures across different potential pollution sources from questionnaire responses at beginning of monitor deployment.

Questionnaire responses	Number of days monitored	Arithmetic mean (SD) exposure	$PM_{2.5}$ exposure (µg/m^3) Minimum exposure	Median exposure	Maximum exposure
Location of main cooker in home					
Inside	800	28.2 (21.4)	3.7	22.7	213.9
Outside	286	27.9 (17.2)	7.1	24.3	160.9
No response	23	21.1 (13.8)	6.3	14.5	51.0
Does the participant usually take part in cooking					
No	204	27.8 (22.3)	5.5	23.0	213.9
Yes	901	28.1 (19.8)	3.7	23.0	197.7
No response	4	10.2 (2.5)	8.4	9.3	13.8
Does the participant live near a busy road					
No	278	29.1 (21.9)	5.8	23.5	213.9
Yes	808	27.5 (19.7)	3.7	22.7	197.7
No response	23	31.9 (20.5)	7.2	25.8	78.8
Does the participant have pets					
No	662	27.0 (18.2)	3.7	22.9	160.9
Yes	447	29.3 (23.0)	4.4	22.9	213.9
Can the participant smell or see waste burning while at home					
No	362	30.9 (26.4)	4.4	23.4	213.9
Yes	731	26.6 (16.3)	3.7	22.8	125.3
Don't know	16	22.3 (16.6)	5.8	14.6	64.4
Does the participant sometimes breathe in smoke from bush fires					
No	555	28.8 (21.1)	4.4	24.0	197.7
Yes	538	27.4 (19.5)	3.7	22.4	213.9
Don't know	16	16.3 (11.1)	8.2	13.2	47.2
Does anyone in the home use cleaning spray					
No	612	30.7 (22.1)	4.8	25.1	213.9
Yes	437	25.0 (17.9)	3.7	20.3	197.7
Don't know	60	21.5 (10.6)	6.3	20.0	51.0
Does anyone in the home use insecticide spray					
No	102	29.6 (22.6)	8.4	22.0	140.5
Yes	166	24.6 (18.0)	5.6	19.1	135.8
Don't know	17	17.6 (7.5)	7.5	16.3	32.1
Does anyone in the home use deodorant spray					
No	314	31.6 (23.3)	4.4	24.5	197.7
Yes	718	26.8 (19.3)	3.7	22.4	213.9
Don't know	77	24.1 (12.8)	5.8	21.6	78.8
Does anyone in the home use mosquito coils					
No	405	27.8 (20.8)	4.8	22.5	213.9
Yes	680	28.1 (20.3)	3.7	23.3	197.7
Don't know	24	26.1 (9.5)	6.3	24.7	41.8

Questionnaire responses	Number of days monitored	PM2.5 exposure (µg/m³) Arithmetic mean (SD) exposure	Minimum exposure	Median exposure	Maximum exposure
Does anyone in the home burn incense					
No	753	28.3 (21.2)	4.8	22.6	213.9
Yes	272	28.5 (19.5)	3.7	24.5	197.7
Don't know	84	22.5 (12.5)	4.8	19.6	54.4
Does anyone in the home burn crop residue					
No	715	27.1 (19.5)	4.8	22.3	173.5
Yes	312	30.1 (22.2)	3.7	25.2	213.9
Don't know	82	26.7 (18.5)	5.8	21.0	118.4
Does anyone use air conditioning in the home					
No	850	28.8 (20.9)	3.7	23.2	213.9
Yes	243	25.4 (17.9)	4.4	22.5	197.7
Don't know	16	20.9 (14.7)	6.3	14.1	51.0
Can the participant smell or see brick burning while at home					
No	835	27.3 (19.6)	3.7	22.5	173.5
Yes	240	29.3 (22.0)	4.4	23.9	213.9
Don't know	34	33.5 (23.7)	5.8	33.3	118.4
Is there someone in the house who is a smoker					
No Smokers	880	27.1 (19.3)	3.7	22.5	197.7
Presence of smokers	211	31.3 (23.9)	4.8	24.4	213.9
No Response	18	29.0 (15.0)	10.7	24.1	64.4
Main cooker in the house					
Electric	442	24.5 (18.4)	3.7	20.3	173.5
Gas	284	27.2 (20.5)	4.4	22.0	197.7
Gas and biomass	240	28.1 (15.8)	7.4	24.5	124.4
Coal or wood (biomass)	71	39.3 (26.2)	10.1	34.5	160.9
Kerosene	23	33.2 (16.1)	13.2	28.5	87.6
Open fire and biomass	41	46.7 (33.0)	15.9	39.0	213.9
Not reported	8	27.9 (9.9)	6.3	29.4	41.8
Lighting in the house					
Electric	365	24.6 (18.0)	3.7	20.0	173.5
Candle	183	27.7 (18.3)	7.1	24.1	124.4
Candle and mixed	92	34.0 (14.7)	7.2	32.4	74.1
Kerosene lamp and mixed	239	29.4 (18.1)	4.8	25.0	131.9
Recharge, solar lamp or torch	198	28.9 (26.6)	4.4	23.2	213.9
Not reported	32	33.2 (31.2)	5.8	24.0	125.3

Table S10.3: Summary statistics by daily exposure by participant for mean, minimum, median and maximum $PM_{2.5}$ exposures across different potential pollution sources from diary responses.

Diary responses	Number of days monitored	$PM_{2.5}$ daily exposure (µg/m^3) Arithmetic mean (SD) exposure	Minimum exposure	Median exposure	Maximum exposure
**Did the participant use the backpack cover on monitoring day**					
Not used	995	28.4 (21.1)	3.7	23.1	213.9
Used	114	24.7 (11.8)	4.8	21.8	71.4
**Was the participant near any of these pollution sources on monitoring day**					
Waste burning site					
No	899	27.1 (19.1)	3.7	22.3	197.7
Yes	210	31.6 (24.4)	7.0	25.3	213.9
Construction site					
No	1015	27.4 (18.9)	3.7	22.8	197.7
Yes	94	33.7 (31.0)	5.8	25.2	213.9
Someone smoking					
No	952	27.1 (19.4)	4.4	22.3	197.7
Yes	157	33.3 (24.4)	3.7	27.0	213.9
Brick burning site					
No	1080	27.8 (20.4)	3.7	22.7	213.9
Yes	29	34.9 (14.9)	15.3	32.0	66.3
Bush fire					
No	1077	27.9 (20.3)	3.7	22.9	213.9
Yes	32	30.4 (18.7)	4.4	25.5	88.8
Electricity generator					
No	1054	28.0 (20.6)	3.7	22.9	213.9
Yes	55	27.5 (11.9)	10.7	24.3	68.6
Participant wasn't near any of these pollution sources					
Noted sources	419	30.7 (22.3)	3.7	24.9	213.9
No noted sources	690	26.3 (18.8)	4.8	22.0	197.7
**Did the participant undertake any of these activities on monitoring day**					
Cooking					
No	817	26.2 (18.9)	3.7	21.6	197.7
Yes	292	32.8 (23.0)	8.1	26.9	213.9
Contact with animals					
No	945	27.0 (18.6)	3.7	22.3	197.7
Yes	164	33.2 (27.5)	4.4	26.3	213.9
Smoking					
No	1104	27.9 (20.3)	3.7	22.9	213.9
Yes	5	29.4 (9.5)	16.2	28.5	39.0
Participant didn't undertake any of these activities					
Noted pollution activities	396	31.9 (22.2)	4.4	26.1	213.9
No noted activity	713	25.7 (18.8)	3.7	21.4	197.7

Table S10.4: Summary statistics by daily exposure by participant for mean, minimum, median and maximum daily $PM_{2.5}$ exposures across other variables.

Other variables	Number of days monitored	$PM_{2.5}$ daily exposure (µg/m^3) Arithmetic mean (SD) exposure	Minimum exposure	Median exposure	Maximum exposure
Participant commute mode on monitoring day					
Motorised	358	25.8 (19.3)	5.5	20.6	197.7
Walk	507	30.8 (21.5)	4.8	25.5	213.9
Mixed mode	96	25.8 (11.8)	7.9	22.8	78.8
No commute	98	23.5 (20.5)	3.7	20.6	160.9
Unclassified	50	27.0 (23.3)	6.2	23.6	172.6
Participant gender					
Female	663	28.0 (22.6)	4.4	22.3	213.9
Male	446	27.9 (16.2)	3.7	24.1	123.4
Participant school ground surface					
Loose dirt	648	30.9 (20.3)	3.7	25.6	213.9
Packed dirt	65	23.4 (10.6)	6.2	19.6	67.5
Broken paving	74	35.3 (29.1)	5.8	26.1	125.3
Paved	322	21.2 (17.0)	4.4	17.5	197.7
Day of week					
Monday	288	28.6 (22.7)	7.1	22.3	197.7
Tuesday	279	27.8 (18.0)	3.7	23.1	125.3
Wednesday	274	28.1 (18.6)	5.6	24.4	172.6
Thursday	268	27.2 (21.5)	4.4	22.8	213.9

11. Additional mixed effects models for sensitivity analysis

Tables S11.1 to S11.3 present mixed effects models for mean daily $PM_{2.5}$ exposure specifically in the main microenvironments, 'at home', 'at school' and 'commuting'. This is provided as a secondary analysis to complement the main mixed effects model (Table 2). The results largely present similar statistically significant determinants to the main mixed effects model, however some differences are highlighted below.

The main difference on determinants for the 'at home' mixed effects model (S11.1) is that the location of the cooker was not found to be a significant determinant of 'at home' exposure. Lighting type was also insignificant determinant for 'at home' exposure, however there was a weak association with kerosene lamps having higher exposure compared to electric only lighting ($p = 0.073$). Additionally, due to the variation in the time spent at home each day, a variable for time was included in the model. This found for each additional hour a participant spent at home increased 'at home' $PM_{2.5}$ exposure by 1.7% (95% confidence interval (CI): 0.4%; 3·0%).

For 'at school' exposures there were some different statistically significant determinants highlighted from the main mixed effects model (S11.2). Children who indicated they were near a waste burning site during the day had an average of 10.3% (CI: 1·4%; 20·0%) higher exposure, while children who indicated they were near a construction site had an average of 16.3% (CI: -27·2%; -3·7%) lower exposure. It is unclear as to why there was lower exposure with children near constructions sites and further research is needed to understand this effect. However, as our participant diary did not specify at what time of day participants were around these sites (SI.5), it is difficult to ascertain if these variables directly related to the school location. Male participants were found to have on average 14.3% (CI: 3.6%; 26.3%) higher exposure at school compared to females. There needs to be further research undertaken to identify what caused this difference in exposure between genders.

For 'commuting' exposures, there was no significant differences to the main mixed effects model apart from day of the week (S11.3). As the model only included those participants who commuted, 'no commute' and 'not reported' categories could not be included in the model. The model highlighted that an additional hour spent commuting resulted in an average of 20.9% (CI: 14.3%; 27.4%) higher $PM_{2.5}$ 'commuting' exposure.

The day of the week variable found significantly lower exposures on Tuesday, Wednesday and Thursday compared to Monday for the 'at home' model. This can be explained as the monitors were provided to the children on Monday morning and therefore morning 'at home' exposures (midnight to 9 am) on this day were missing, which would typically result in low exposure levels as there is likely to be fewer sources of $PM_{2.5}$ during this time. Conversely, to the 'at home' mixed effects model, the 'at school' and 'commuting' model found significantly higher exposures on Tuesday, Wednesday and Thursday compared to Monday. This is due to the monitoring on Mondays missed the morning commute and morning exposure levels at school which could be high.

We also conducted a sensitivity analysis to the main model, removing Monday exposures (S11.4); as backpacks deployed on this day missed the midnight to 9 am time period. The only statistically significant difference which was found in this model was that average daily male $PM_{2.5}$ exposures were 9.2% (CI: 0.5%; 18.9%) higher compared to female.

While there were some differences highlighted in the individual microenvironment mixed effects models, the main determinants of high daily $PM_{2.5}$ exposure were similar, such as smokers in the home, use of biomass for cooking and loose dirt on school grounds. However, it is important to note there is difficulty with interpreting these microenvironment models as each participant spent a different amount of time in each microenvironment. Furthermore, an additional 61 days (6%) of data were removed from this analysis as some participants did not have GPS coordinates to provide microenvironment segmentation. Therefore, these models should be interpreted with caution.

Table S11.1: Fixed effects determinants from mixed effects model for children's logged daily personal $PM_{2.5}$ exposure while 'at home'.

Fixed effect determinants	Categorical comparison	Reference group	Change %	95% CI	*p*-value
Near waste burning site	Yes	No	-4.5	-13.5, 6.0	0.374
Near construction site	Yes	No	9.4	-6.0, 27.5	0.256
Help with cooking	Yes	No	4.7	-5.6, 16.8	0.400
Contact with animals	Yes	No	-5.8	-16.2, 6.3	0.329
Cooker location	Outside	Inside	-8.6	-19.7, 3.9	0.189^{+}
	Not reported	Inside	-29.3	-51.6, 4.0	0.088*
Cooker type	**Gas**	**Electric**	**16.5**	**0.3, 34.9**	**<0.05**
	Gas and biomass	Electric	10.9	-7.6, 33.0	0.283
	**Coal or wood (biomass)**	**Electric**	**67.7**	**29.4, 119.8**	**<0.001**
	**Kerosene**	**Electric**	**49.2**	**1.9, 118.7**	**<0.05^{+}**
	**Open fire and biomass**	**Electric**	**54.7**	**13.1, 113.5**	**<0.01**
	Not reported	Electric	21.9	-35.0, 128.1	0.551
Lighting use	Candle	Electric only	8.3	-9.1, 30.0	0.395
	Candle and mixed	Electric only	11.2	-7.0, 33.5	0.269
	Kerosene lamp and mixed	Electric only	24.6	-1.1, 56.9	0.073*$^{+}$
	Recharge, solar lamp or torch	Electric only	14.3	-2.0, 34.1	0.106^{+}
	Not reported	Electric only	38.0	-2.4, 95.8	0.082*
Smoking at home	**Presence of smokers**	**No smokers**	**25.3**	**9.1, 44.2**	**<0.01**
	Not reported	No smokers	10.1	-28.6, 69.7	0.673
Gender	Male	Female	-6.2	-15.8, 4.6	0.262
Lives near busy road	No	Yes	-2.9	-14.2, 10.4	0.661
	Not reported	Yes	20.3	-18.6, 79.5	0.378
Day of the week	**Tuesday**	**Monday**	**-19.2**	**-29.0, -8.3**	**<0.01^{+}**
	**Wednesday**	**Monday**	**-16.5**	**-27.0, -4.9**	**<0.01^{+}**
	**Thursday**	**Monday**	**-23.9**	**-33.3, -13.4**	**<0.001^{+}**
**Mean daily air temperature (°C)**			**3.4**	**0.7, 5.7**	**<0.01**
**Mean daily relative humidity (%)**			**-0.7**	**-1.3, -0.1**	**<0.05^{+}**
**Mean daily wind speed (ms^{-1})**			**-12.5**	**-15.9, -8.9**	**<0.001**
**Time spent at home (hr)**			**1.7**	**0.4, 3.0**	**<0.05**

Results < 0·05 are shown in bold, * >0·05 but <0·10. 'change %' calculated by (exp(effect estimate) − 1) × 100%.
+ shows different statistical significance from main model

Table S11.2: Fixed effects determinants from mixed effects model for children's logged daily personal PM$_{2.5}$ exposure while 'at school'.

Fixed effect determinants	Categorical comparison	Reference group	Change %	95% CI	*p*-value
**Near waste burning site**	**Yes**	**No**	**10.3**	**1.4, 20.0**	**<0.05**$^{+}$
**Near construction site**	**Yes**	**No**	**-16.3**	**-27.2, -3.7**	**<0.05**$^{+}$
**Gender**	**Male**	**Female**	**14.3**	**3.6, 26.3**	**<0.01**$^{+}$
School ground surface	**Packed dirt**	**Loose dirt**	**-37.1**	**-49.2, -22.2**	**<0.001**$^{+}$
	Broken paving	Loose dirt	-11.3	-36.7, 22.2	0.476
	**Paved**	**Loose dirt**	**-40.5**	**-52.3, -26.1**	**<0.001**
Day of the week	**Tuesday**	**Monday**	**25.9**	**12.9, 40.7**	**<0.001**$^{+}$
	**Wednesday**	**Monday**	**21.7**	**9.3, 35.5**	**<0.001**$^{+}$
	**Thursday**	**Monday**	**25.9**	**13.1, 40.3**	**<0.001**$^{+}$
**Mean daily air temperature (°C)**			**5.3**	**2.9, 7.6**	**<0.001**
**Mean daily relative humidity (%)**			**0.6**	**0.1, 1.1**	**<0.05**$^{+}$
**Mean daily wind speed (ms^{-1})**			**-4.6**	**-7.8, -1.1**	**<0.01**
Time spent at school (hr)			0.5	-1.6, 2.6	0.660

Results < 0·05 are shown in bold, * >0·05 but <0·10. 'change %' calculated by (exp(effect estimate) − 1) × 100%. + shows different statistical significance from main model

Table S11.3: Fixed effects determinants from mixed effects model for children's logged daily personal PM$_{2.5}$ exposure while 'commuting'.

Fixed effect determinants	Categorical comparison	Reference group	Change %	95% CI	*p*-value
Near waste burning site	Yes	No	-1.4	-10.1, 8.2	0.760
Near construction site	Yes	No	-3.0	-15.9, 12.0	0.682
Commute type	Motorised	Walk	7.8	-3.0, 19.5	0.160
	Mixed mode	Walk	-3.3	-15.4, 10.4	0.623
Gender	Male	Female	-5.1	-13.5, 4.1	0.267
Day of the week	**Tuesday**	**Monday**	**25.6**	**14.1, 38.5**	**<0.001**$^{+}$
	**Wednesday**	**Monday**	**22.5**	**11.3, 35.2**	**<0.001**$^{+}$
	**Thursday**	**Monday**	**20.6**	**9.3, 33.5**	**<0.001**$^{+}$
**Mean daily air temperature (°C)**			**3.8**	**1.6, 6.1**	**<0.001**
Mean daily relative humidity (%)			-0.2	-0.7, 0.3	0.509
**Mean daily wind speed (ms^{-1})**			**-6.9**	**-10.4, -3.1**	**<0.001**
**Time spent commuting (hr)**			**20.9**	**14.3, 27.4**	**<0.001**

Results < 0·05 are shown in bold, * >0·05 but <0·10. 'change %' calculated by (exp(effect estimate) − 1) × 100%.
+ shows different statistical significance from main model

Table S11.4: Fixed effects determinants from mixed effects model for children's logged daily personal $PM_{2.5}$ exposure excluding Monday exposure measurements.

Fixed effect determinants	Categorical comparison	Reference group	Change %	95% CI	*p*-value
Near waste burning site	Yes	No	3.1	-5.2, 12.8	0.497
Near construction site	Yes	No	8.5	-4.5, 23.1	0.217
Help with cooking	Yes	No	4.0	-5.2, 14.5	0.425
Contact with animals	Yes	No	2.0	-7.7, 13.2	0.707
Commute type	Motorised	Walk	1.2	-8.4, 11.3	0.814
	Mixed mode	Walk	-3.7	-14.9, 8.2	0.555
	**No commute**	**Walk**	**-18.8**	**-29.0, -7.4**	**<0.01**
	Not recorded	Walk	-11.9	-25.0, 2.9	0.125
Cooker location	**Outside**	**Inside**	**-15.3**	**-23.4, -6.5**	**<0.01**
	Not reported	Inside	-7.7	-31.6, 24.5	0.618
Cooker type	**Gas**	**Electric**	**16.8**	**3.9, 31.1**	**<0.05**
	Gas and biomass	Electric	12.7	-2.4, 30.2	0.123
	**Coal or wood (biomass)**	**Electric**	**28.2**	**4.3, 58.7**	**<0.05**
	Kerosene	Electric	19.4	-11.4, 60.8	0.265
	**Open fire and biomass**	**Electric**	**32.8**	**3.7, 71.1**	**<0.05**
	Not reported	Electric	46.2	-10.9, 138.5	0.150
Lighting use	Candle	Electric only	10.9	-3.2, 27.6	0.158
	Candle and mixed	Electric only	10.0	-4.3, 26.9	0.206
	**Kerosene lamp and mixed**	**Electric only**	**26.3**	**5.2, 51.2**	**<0.05**
	**Recharge, solar lamp or torch**	**Electric only**	**15.9**	**2.7, 31.3**	**<0.05**
	Not reported	Electric only	3.7	-21.8, 38.6	0.811
Smoking at home	**Presence of smokers**	**No smokers**	**26.4**	**13.5, 40.8**	**<0.001**
	Not reported	No smokers	5.6	-25.1, 47.9	0.766
Gender	**Male**	**Female**	**9.2**	**0.5, 18.9**	**<0.05**+
Lives near busy road	No	Yes	3.3	-6.0, 13.9	0.522
	Not reported	Yes	13.2	-17.0, 55.8	0.463
School ground surface	Packed dirt	Loose dirt	-2.6	-18.7, 17.1	0.787
	Broken paving	Loose dirt	34.8	1.4, 80.7	0.056*
	**Paved**	**Loose dirt**	**-34.0**	**-45.7, -18.9**	**<0.001**
Day of the week	Wednesday	Tuesday	0.5	-5.8, 7.3	0.877
	Thursday	Tuesday	-2.1	-8.2, 4.7	0.523
**Mean daily air temperature (°C)**			**5.3**	**2.9, 7.4**	**<0.001**
Mean daily relative humidity (%)			-0.2	-0.7, 0.3	0.451
**Mean daily wind speed (ms^{-1})**			**-7.6**	**-10.7, -4.3**	**<0.001**

Results < 0·05 are shown in bold, * >0·05 but <0·10. 'change %' calculated by (exp(effect estimate) − 1) × 100%.
+ shows different statistical significance from main model

12. References

1 DEFRA. Site information Honor Oak Park- Defra, UK. 2019. https://uk-air.defra.gov.uk/networks/site-info?site_id=HP1 (accessed June 27, 2021).

2 Dalumpines R, Scott DM. Making mode detection transferable: extracting activity and travel episodes from GPS data using the multinomial logit model and Python. *Transp Plan Technol* 2017; **40**: 523–39.

3 Finazzi F, Paci L. Quantifying personal exposure to air pollution from smartphone-based location data. *Biometrics* 2019; : 13100.

4 van Dijk J. Identifying activity-travel points from GPS-data with multiple moving windows. *Comput Environ Urban Syst* 2018; **70**: 84–101.

5 Gong H, Chen C, Bialostozky E, Lawson CT. A GPS/GIS method for travel mode detection in New York City. *Comput Environ Urban Syst* 2012; **36**: 131–9.

6 Hijmans R. geosphere: Spherical Trigonometry. 2019. https://CRAN.R-project.org/package=geosphere.

7 Grolemund G, Wickham H. Dates and Times Made Easy with lubridate. *J Stat Softw* 2011; **40**: 1–25.

8 Zeileis A, Grothendieck G. zoo: S3 Infrastructure for Regular and Irregular Time Series. *J Stat Softw* 2005; **14**: 1–27.

9 Birant D, Kut A. ST-DBSCAN: An algorithm for clustering spatial–temporal data. *Intell Data Min* 2007; **60**: 208–21.

10 Brusilovskiy E, Klein LA, Salzer MS. Using global positioning systems to study health-related mobility and participation. *Soc Sci Med* 2016; **161**: 134–42.

11 Carslaw D. worldmet: Import Surface Meteorological Data from NOAA Integrated Surface Database (ISD). 2019. https://CRAN.R-project.org/package=worldmet.
